# Dihydroorotase MoPyr4 is required for development, pathogenicity, and autophagy in rice blast fungus

**DOI:** 10.1186/s12964-024-01741-4

**Published:** 2024-07-15

**Authors:** Jing-Yi Wang, Ying-Ying Cai, Lin Li, Xue-Ming Zhu, Zi-Fang Shen, Zi-He Wang, Jian Liao, Jian-Ping Lu, Xiao-Hong Liu, Fu-Cheng Lin

**Affiliations:** 1 Xianghu Laboratory, State Key Laboratory for Managing Biotic and Chemical Treats to the Quality and Safety of Agro-products, Institute of Biotechnology, Zhejiang University, Hangzhou, 310058 China; 2 Xianghu Laboratory, State Key Laboratory for Managing Biotic and Chemical Treats to the Quality and Safety of Agro-products, Institute of Plant Protection and Microbiology, Zhejiang Academy of Agricultural Sciences, Hangzhou, 310021 China; 3 College of Life Sciences, Zhejiang University, Hangzhou, 310058 China

**Keywords:** *Magnaporthe oryzae*, Dihydroorotase, Pyrimidine nucleotide biosynthesis, Pathogenicity, Autophagy

## Abstract

Dihydroorotase (DHOase) is the third enzyme in the six enzymatic reaction steps of the endogenous pyrimidine nucleotide de novo biosynthesis pathway, which is a metabolic pathway conserved in both bacteria and eukaryotes. However, research on the biological function of DHOase in plant pathogenic fungi is very limited. In this study, we identified and named MoPyr4, a homologous protein of *Saccharomyces cerevisiae* DHOase Ura4, in the rice blast fungus *Magnaporthe oryzae* and investigated its ability to regulate fungal growth, pathogenicity, and autophagy. Deletion of *MoPYR4* led to defects in growth, conidiation, appressorium formation, the transfer and degradation of glycogen and lipid droplets, appressorium turgor accumulation, and invasive hypha expansion in *M. oryzae*, which eventually resulted in weakened fungal pathogenicity. Long-term replenishment of exogenous uridine-5’-phosphate (UMP) can effectively restore the phenotype and virulence of the Δ*Mopyr4* mutant. Further study revealed that MoPyr4 also participated in the regulation of the Pmk1-MAPK signaling pathway, co-localized with peroxisomes for the oxidative stress response, and was involved in the regulation of the Osm1-MAPK signaling pathway in response to hyperosmotic stress. In addition, MoPyr4 interacted with MoAtg5, the core protein involved in autophagy, and positively regulated autophagic degradation. Taken together, our results suggested that MoPyr4 for UMP biosynthesis was crucial for the development and pathogenicity of *M. oryzae*. We also revealed that MoPyr4 played an essential role in the external stress response and pathogenic mechanism through participation in the Pmk1-MAPK signaling pathway, peroxisome-related oxidative stress response mechanism, the Osm1-MAPK signaling pathway and the autophagy pathway.

**Supplementary Information:**

The online version contains supplementary material available at 10.1186/s12964-024-01741-4.

## Introduction

Pyrimidine nucleotides are essential for the normal physiological function of cells because they play important roles not only in the biosynthesis of genetic material, but also in the metabolism of various substances in intracellular physiological and biochemical processes [1, 2]. There are two pathways for pyrimidine nucleotide biosynthesis in vivo: the de novo and salvage pathways. The endogenous pyrimidine nucleotide de novo biosynthesis pathway uses glutamine (Gln), aspartic acid (Asp), phosphoribosyl pyrophosphate (PRPP), and carbon dioxide (CO_2_) as raw materials and synthesizes pyrimidine nucleotides through six conserved enzymatic reaction steps [3]. Uridine-5’-phosphate (UMP) is the first pyrimidine nucleotide to be synthesized in vivo through a de novo synthesis pathway, and it acts as a precursor for all other pyrimidine nucleotides that are subsequently synthesized [4]. Dihydroorotase (DHOase) is the third enzyme of the six enzymatic reaction steps, dehydrating and intramolecularly rearranging intermediate carbamyl aspartate to form cyclized dihydroorotic acid, thereby synthesizing the pyrimidine ring [5]. In lower eukaryotes such as yeast and plants, DHOase is a mono-functional enzyme encoded by a single gene. In some higher eukaryotes, DHOase is fused into a polypeptide chain with the first enzyme carbamylphosphate synthetase (CPSase) and the second enzyme aspartate transcarbamylase (ATCase), and functions in the form of carbamoyl-phosphate synthetase, aspartate transcarbamoylase, and dihydroorotase (CAD), which are multifunctional enzymes that are more conducive to the synthesis of products at a steady rate [6]. In recent years, studies in mammalian cells have shown that DHOase can be used as a new antitumor drug screening target [7], and the inhibitor that targets DHOase has potential for use in anticancer drug development [8]. In plant pathogens, a study that analyzed the relative expression profile of pyrimidine biosynthesis genes during the infection of diploid potatoes by *Phytophthora infestans* revealed that the expression of DHOase was upregulated in biotrophs and downregulated in necrotrophic plants, first suggesting the importance of DHOase in the early infection stage when pathogenic fungi replicate rapidly [9].

The plant pathogen *Magnaporthe oryzae* is a filamentous ascomycete fungus that causes rice blast, one of the most destructive diseases in rice production globally [10, 11]. *M. oryzae* has a typical fungal development pattern and infection cycle and can be genetically manipulated at the molecular biological level; therefore, it has become a model for studying the pathogenic mechanism of pathogenic fungi [12, 13]. The life cycle of *M. oryzae* includes both sexual and asexual stages, and its asexual state completes the entire infection cycle under natural conditions [14]. The conidia are induced to secrete mucilage upon attaching to the hydrophobic waxy surface of plant leaves to improve adhesion to the leaf surface [15]. Subsequently, germ tubes are generated from the conidia under certain humidity conditions within 2 h which expand into a hook-like body within 4 to 6 h. Then, the hook-like body further grows to form a specific invasive structure called the ‘appressorium’, which matures within 24 h. During the process of the appressoria development, the conidia undergo autophagic cell death and eventually collapse, and nutrients in the conidia, such as glycogen and lipid droplets, are degraded and transferred to the appressoria to provide energy [16]. After continuous decomposition and synthesis of nutrients inside the appressoria, a large amount of glycerol accumulates, resulting in high osmotic pressure. The dense melanin layer between the inner plasma membrane and the cell wall inside the appressoria converts the osmotic pressure into turgor pressure up to 8.0 MPa [17], which is ultimately transformed into mechanical force with the formation of a ‘penetration peg’. The penetration peg penetrates the cuticle of the leaf and invades the epidermal cells of the host plant. Soon afterward, invasive hyphae undergo further infection through continuous mitosis and the multibranched invasive hyphae quickly expand to adjacent cells [18].

The above pathogenetic processes of *M. oryzae* depend on the precise regulation of multiple signaling pathways [19]. One of the mitogen-activated protein kinase (MAPK)-mediated pathways, the Pmk1-MAPK signaling pathway, is mainly composed of Mst11-Mst7-Pmk1 and the scaffold protein Mst50, which regulates not only appressorium formation, but also penetration peg differentiation and invasive hypha expansion in host plants [19–21]. Another MAPK signaling pathway, Osm1-MAPK, is mainly composed of Ssk2-Pbs2-Osm1 and the scaffold protein Mst50, which regulate intracellular osmotic pressure through the recognition of extracellular high osmotic stress signals after pathogenic fungi invade host plants [19].

Autophagy is an intracellular degradation pathway in eukaryotes that is highly conserved from yeast to mammals. Under starvation or certain stress conditions, cytosolic contents such as organelles, protein aggregations and pathogens are enclosed as substrates by several double-membraned vesicles called autophagosomes and then transported to vacuoles (fungi and plants) or lysosomes (mammals) for degradation and recycling to meet the requirements of the intracellular nutrient supply and metabolic cycle [22]. Currently, 18 autophagy core proteins encoded by autophagy-related genes (ATG), which are involved in autophagosome formation have been identified in *Saccharomyces cerevisiae*, and their functions and interaction patterns are highly conserved in eukaryotes [23, 24]. Five systems have been identified based on their functions: the Atg1-Atg13-Atg17 initiation complex, the Atg9-Atg2-Atg18 membrane cycling system, the phosphatidylinositol-3-kinase (PI3K) complex, the Atg12-Atg5-Atg16 ubiquitin system, and the Atg8-PE ubiquitin system, which combines Atg8 with phosphatidylethanolamine (PE) [25]. In recent years, several reports have shown that autophagy not only regulates the growth of plant pathogenic fungi, but also plays a crucial role in fungal pathogenesis [26]. Deletion of most yeast ATG-homologous autophagy core genes in *M. oryzae* not only leads to defects in conidiation, conidial germination, and turgor accumulation in the appressorium, but also results in complete loss of pathogenicity [27, 28]. In other filamentous fungi, including *Colletotrichum spp*., *Botrytis cinerea*, *Sordaria macrospora*, *Phytophthora sojae*, and *Aspergillus niger*, the results of the reduction or loss of pathogenicity after the knockout of autophagy core genes also suggested the conserved and universal role of autophagy in the pathogenicity of plant pathogenic fungi [29].

Although the importance of DHOase has been preliminarily studied in infection process of *P. infestans*, its biological function in pathogenic fungi and its roles in pathogenicity-related signaling pathways have not been systematically investigated. In this study, DHOase was identified and named MoPyr4 in model pathogenic fungi *M. oryzae*. We showed the crucial role of MoPyr4 in pyrimidine nucleotide biosynthesis, and revealed that MoPyr4-mediated UMP biosynthesis is essential for the growth, conidiation, appressorium formation, transfer and degradation of glycogen and lipid droplets, appressorium turgor accumulation, invasive hypha expansion, and pathogenicity of *M. oryzae*. Furthermore, MoPyr4 participates in the external stress response and pathogenic mechanism by regulating the Pmk1-MAPK signaling pathway, Osm1-MAPK signaling pathway and oxidative stress response, as well as positively regulates autophagic degradation through interaction with MoAtg5, a core autophagy protein.

## Results

### Identification of DHOase in *M. oryzae*

By using the protein-protein basic local alignment search tool (BLAST) in the NCBI database (https://www.ncbi.nlm.nih.gov/), we found that the protein in *M. oryzae* encoded by MGG_12634 and the DHOase from the de novo pyrimidine nucleotide biosynthesis pathway in *S. cerevisiae* named Ura4 showed 43.67% in amino acid sequence homology. In addition, the homologous protein of the yeast Ura4 in *M. oryzae* was unique. According to the name of the enzyme in yeast, we named the corresponding homologous protein MoPyr4 in *M. oryzae*. Then, we analyzed the homology of DHOase in different fungi and constructed a phylogenetic tree using DNAMAN v.8 and MEGA 7.0.26 software. Homology analysis suggested that MoPyr4 in *M. oryzae* was genetically close to DHOase in most other fungi besides the model fungus *S. cerevisiae*, indicating the evolutionary conservation of DHOase (Figure S1A, B).

To study the biological function of MoPyr4 in *M. oryzae*, the *MoPYR4* gene was knocked out using a high-throughput gene knockout strategy [30]. We constructed the pKO3A knockout vector with the hygromycin resistance gene *HPH* and transferred it into Guy11, the wild-type of *M. oryzae*, by *Agrobacterium tumefaciens*-mediated transformation (ATMT). When the target *MoPYR4* gene was replaced with *HPH* in the Guy11 genome according to the homologous replacement principle, we confirmed the deletion of *MoPYR4* by polymerase chain reaction (PCR) and quantitative real-time polymerase chain reaction (qRT-PCR) and finally obtained the Δ*Mopyr4* mutants (Figure S2A, B). Moreover, to confirm that the phenotypic and physiological differences shown by the Δ*Mopyr4* mutants were indeed caused by deletion of the *MoPYR4* gene, we inserted the *MoPYR4* gene into the genome of the Δ*Mopyr4* mutant via ATMT and obtained the complemented strain Δ*Mopyr4*::*MoPYR4*.

### MoPyr4 is required for the growth and conidiation of *M. oryzae*

**Fig. 1:**
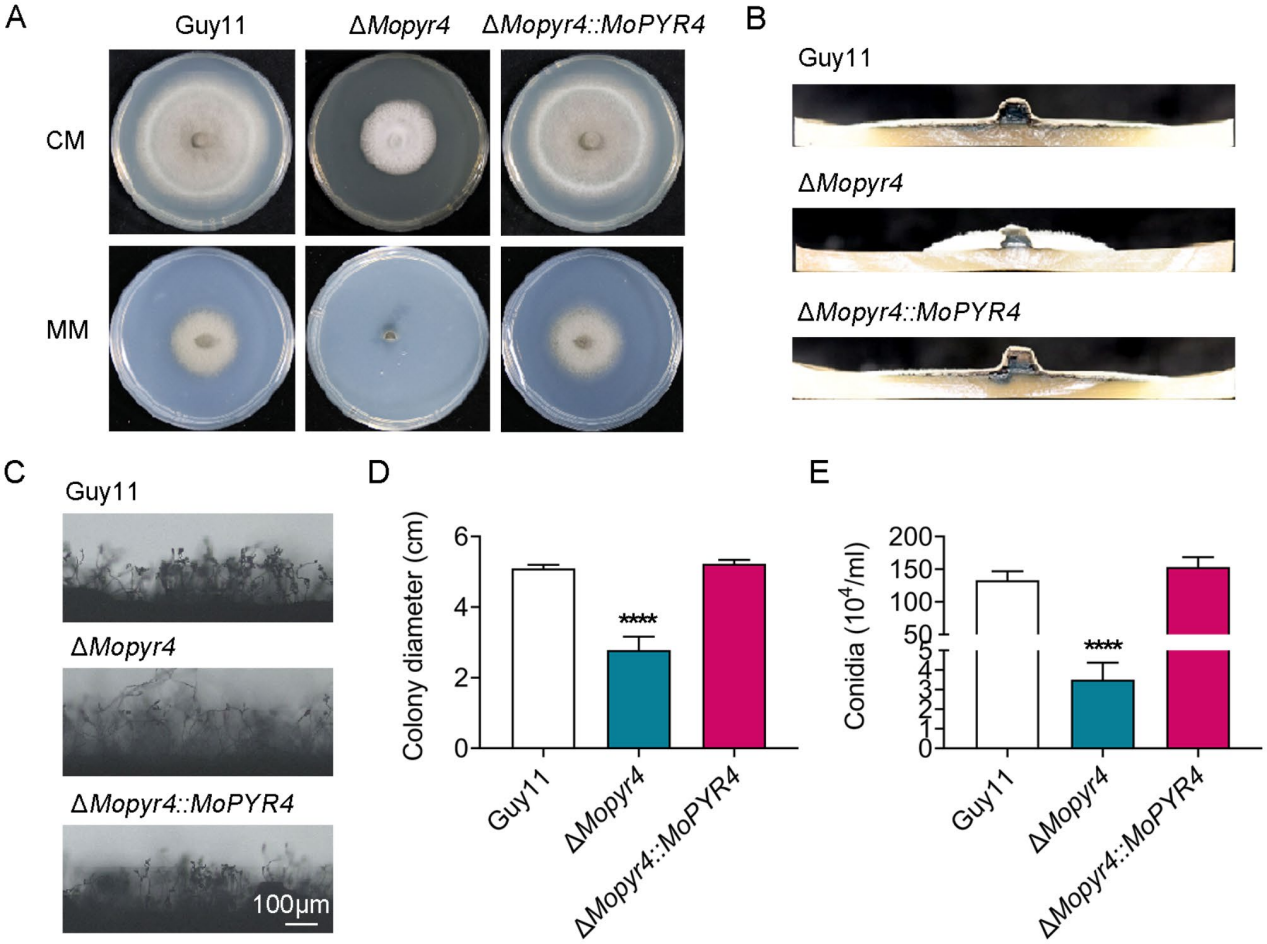
Hyphal growth and conidiation of the Δ*Mopyr4* mutant. (**A**) Hyphal growth of the Guy11, mutant, and complementary strains on CM and MM plates. (**B**) Growth of aerial hyphae of the Guy11, mutant, and complementary strains on CM plates. (**C**) Conidia and conidiophore growth of the Guy11, mutant, and complementary strains. Scale bar, 100 μm. (**D**) Statistical analysis of colony diameters of the Guy11, mutant, and complementary strains on CM plates. The data were calculated from three replicates. Asterisks indicate statistically significant differences (t-test, **** *P* < 0.0001). (**E**) Statistical analysis of the conidiation in the Guy11, mutant, and complementary strains. The data were calculated from three replicates. Asterisks indicate statistically significant differences (t-test, **** *P* < 0.0001)

To determine whether the absence of MoPyr4 affects fungal development, we first measured the colony diameter and found that the growth rate of the Δ*Mopyr4* strain on complete medium (CM) was significantly slower than that of the Guy11 and Δ*Mopyr4*::*MoPYR4* strains (Fig. 1A and D). Moreover, the Δ*Mopyr4* strain could not grow on basic minimal medium (MM) (Fig. 1A). In addition to the colony growth rate, the aerial mycelium of Δ*Mopyr4* was significantly sparser and fluffier (Fig. 1B). Next, we examined the conidia production of each strain. As shown in Fig. 1E, the conidiation of Δ*Mopyr4* was dramatically reduced, displaying an order of magnitude difference from that of Guy11 and Δ*Mopyr4*::*MoPYR4*. The conidia produced by Δ*Mopyr4* were not greater than 3% of those produced by the wild-type and complemented strains [conidiation of Guy11 = (133.0 ± 13.9) × 10^4^ conidia/mL, conidiation of Δ*Mopyr4* = (3.5 ± 0.9) × 10^4^ conidia/mL, conidiation of Δ*Mopyr4*::*MoPYR4 *= (153.3 ± 15.3) × 10^4^ conidia/mL]. Consistent with this result, the conidiophores of Δ*Mopyr4* were sparser than those of Guy11 and Δ*Mopyr4*::*MoPYR4*, and fewer conidia could be observed on the conidiophores of Δ*Mopyr4* (Fig. 1C). The above results indicate that MoPyr4 is essential for the growth and conidiation of *M. oryzae*.

### MoPyr4 is required for the pathogenicity of *M. oryzae*

**Fig. 2:**
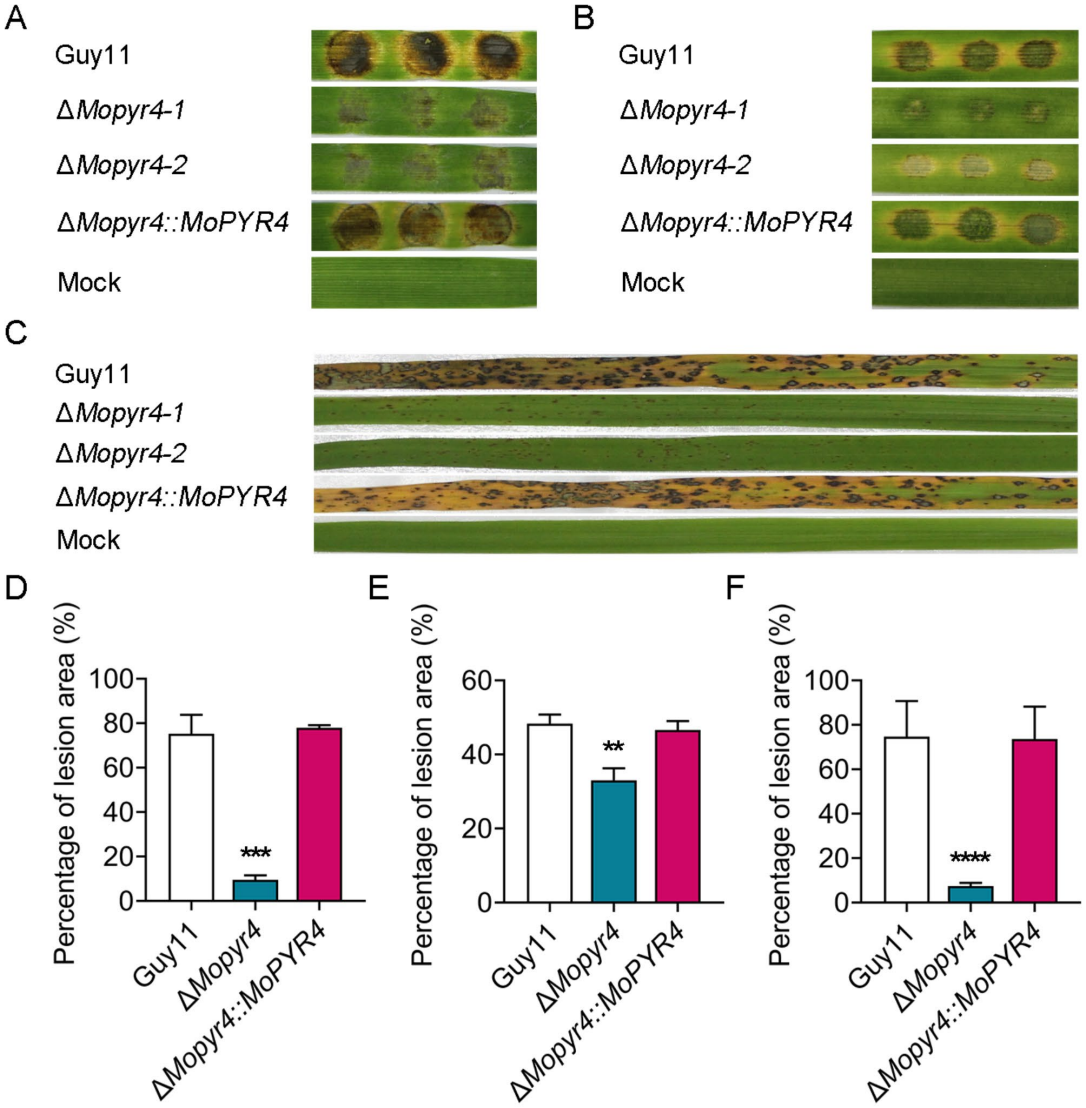
Pathogenicity of the Δ*Mopyr4* mutant. (**A**) Disease spots on detached barley leaves inoculated with mycelial plugs of the Guy11, mutant, and complementary strains. (**B**) Disease symptoms on isolated barley leaves inoculated with conidial suspensions (20 µL, 5 × 10^4^ conidia/mL) of the Guy11, mutant, and complementary strains. **(C)** Disease symptoms of rice seedlings inoculated with conidial suspensions spray (2 mL, 5 × 10^4^ conidia/mL) of the Guy11, mutant, and complementary strains. (**D**, **E**, **F**) Statistical analyses of the percentage of the lesion area per leaf caused by mycelial plugs (**D**), conidial suspensions (**E**), and conidial suspensions spray (**F**) of the Guy11, mutant, and complementary strains. The data in D and E were calculated from three replicates. The data in F were calculated from 15 replicates. Asterisks indicate statistically significant differences (t-test, *** *P* < 0.001, ** *P* < 0.01, **** *P* < 0.0001)

To further investigate the function of MoPyr4 in the pathogenicity of *M. oryzae*, we inoculated the three strains (Guy11, Δ*Mopyr4*, and Δ*Mopyr4*::*MoPYR4*) on two different host plants, barley and rice. First, the same-sized mycelial plugs were cut and inoculated on detached barley leaves. The disease spots on the barley leaves showed that Guy11 and Δ*Mopyr4*::*MoPYR4* both caused severe large brown lesions, and the areas around the lesions on the leaves turned yellow. However, Δ*Mopyr4* caused only small, weak lesions on the barley leaves, and leaf yellowing was not obvious (Fig. 2A and D). Next, the conidial suspensions were inoculated on isolated barley leaves for 4 days (d). The results showed that the disease spots on the barley leaves caused by Guy11 and Δ*Mopyr4*::*MoPYR4* were large and dark, with the leaves turning yellow, and the lesions of adjacent conidial droplets were almost connected to one piece. In contrary, the lesions caused by Δ*Mopyr4* could not expand from one conidial droplet to adjacent droplets, and the percentage of the lesion area was significantly smaller (Fig. 2B and E). To simulate the pathogenic process in the field more accurately, we sprayed a conidial suspension of the three strains on 2-week-old potted rice seedlings (susceptible *Oryza sativa* cv. CO-39). The typical rhombic fusion lesions were caused by the wild-type and complemented strains on the rice seedlings at 7 days post-incubation (dpi), and the leaves were yellow. In contrast, the lesions of the Δ*Mopyr4* mutant were significantly smaller and independent, and the leaves still looked green (Fig. 2C and F). Taken together, the above results indicate that MoPyr4 plays an important role in the virulence of *M. oryzae* on host plants.

### The addition of exogenous UMP restored the growth and conidiation of the Δ*Mopyr4* mutant

**Fig. 3:**
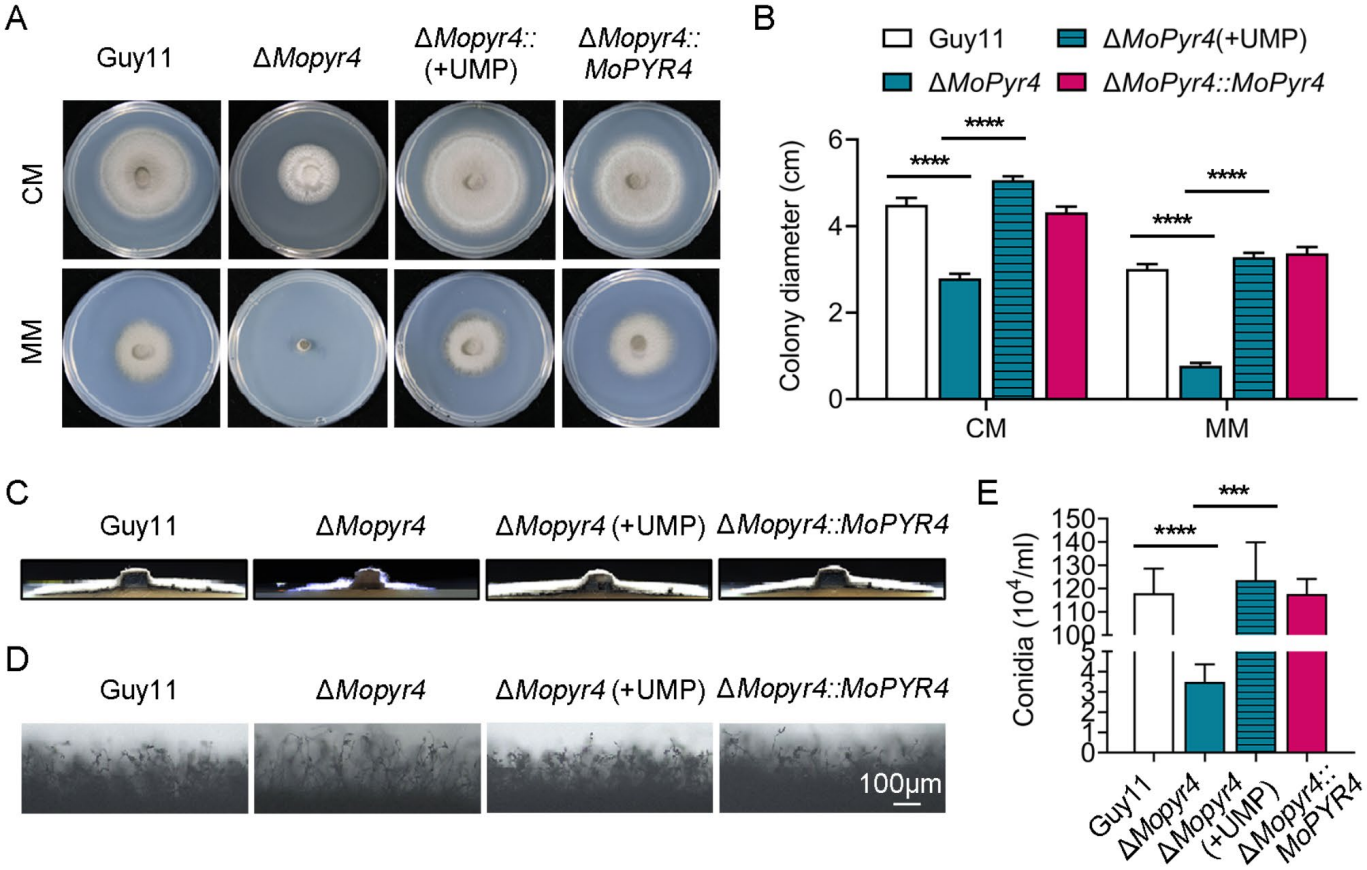
Complementation of the growth of the Δ*Mopyr4* mutant after supplementation with exogenous UMP. (**A**) Hyphal growth of the Guy11, Δ*Mopyr4* mutant, complementary strain, and the Δ*Mopyr4* mutant supplemented with 5 mM exogenous UMP on CM and MM plates. (**B**) Statistical analysis of colony diameters of the Guy11, Δ*Mopyr4* mutant, complementary strain, and the Δ*Mopyr4* mutant supplemented with 5 mM exogenous UMP on CM and MM plates. The data were calculated from three replicates. Asterisks indicate statistically significant differences (t-test, **** *P* < 0.0001). (**C**) Aerial hyphal growth in the Guy11, Δ*Mopyr4* mutant, complementary strain, and the Δ*Mopyr4* mutant supplemented with 5 mM exogenous UMP on CM plates. (**D**) Conidia and conidiophore growth of the Guy11, Δ*Mopyr4* mutant, complementary strain, and the Δ*Mopyr4* mutants supplemented with 5 mM exogenous UMP on CM plates. Scale bar, 100 μm. (**E**) Statistical analysis of conidiation in the Guy11, Δ*Mopyr4* mutant, complementary strain, and the Δ*Mopyr4* mutant supplemented with 5 mM exogenous UMP. The data were calculated from three replicates. Asterisks indicate statistically significant differences (t-test, *** *P* < 0.001, **** *P* < 0.0001)

The enzymes of the de novo pyrimidine nucleotide biosynthesis pathway perform their catalytic functions in turn and first synthesize UMP [4, 5]; therefore, we wondered whether exogenous UMP addition could restore the phenotype of the Δ*Mopyr4* mutants. First, 5 mM UMP was added to the CM, and the colony diameter of each strain was measured at 7 dpi. The results showed that the growth rate of the Δ*Mopyr4* mutant on CM plates significantly increased to the level of the wild-type and complemented strains after the addition of exogenous UMP (Fig. 3A and B). Moreover, the growth of the Δ*Mopyr4* mutants was completely restored on the MM plates supplemented with 5 mM exogenous UMP (Fig. 3A and B). In addition, the aerial mycelia of Δ*Mopyr4* were no longer sparse or fluffy on the CM plates supplemented with UMP, and their growth was restored to levels consistent with those of Guy11 and Δ*Mopyr4*::*MoPYR4* (Fig. 3C). Next, we found that the conidiation of Δ*Mopyr4* recovered to the same level as that of Guy11 or Δ*Mopyr4*::*MoPYR4* after the addition of exogenous UMP (Fig. 3E). Consistent with the results of conidiation recovery, the growth of the conidiophores of Δ*Mopyr4* supplemented with exogenous UMP were consistent with the wild-type and complemented strains (Fig. 3D). These results show that the exogenous UMP restores the growth and conidiation of Δ*Mopyr4*, and confirm that MoPyr4 plays an important role in the de novo pyrimidine nucleotide biosynthesis pathway.

### The addition of exogenous UMP restored the pathogenicity and infection process of the Δ*Mopyr4* mutant

To further explore the reasons for the reduced pathogenic ability of Δ*Mopyr4*, we observed the development of invasive hyphae in barley leaves, and analyzed the recovery of pathogenicity and the appressorium-mediated infection process of Δ*Mopyr4* with exogenous UMP.

**Fig. 4:**
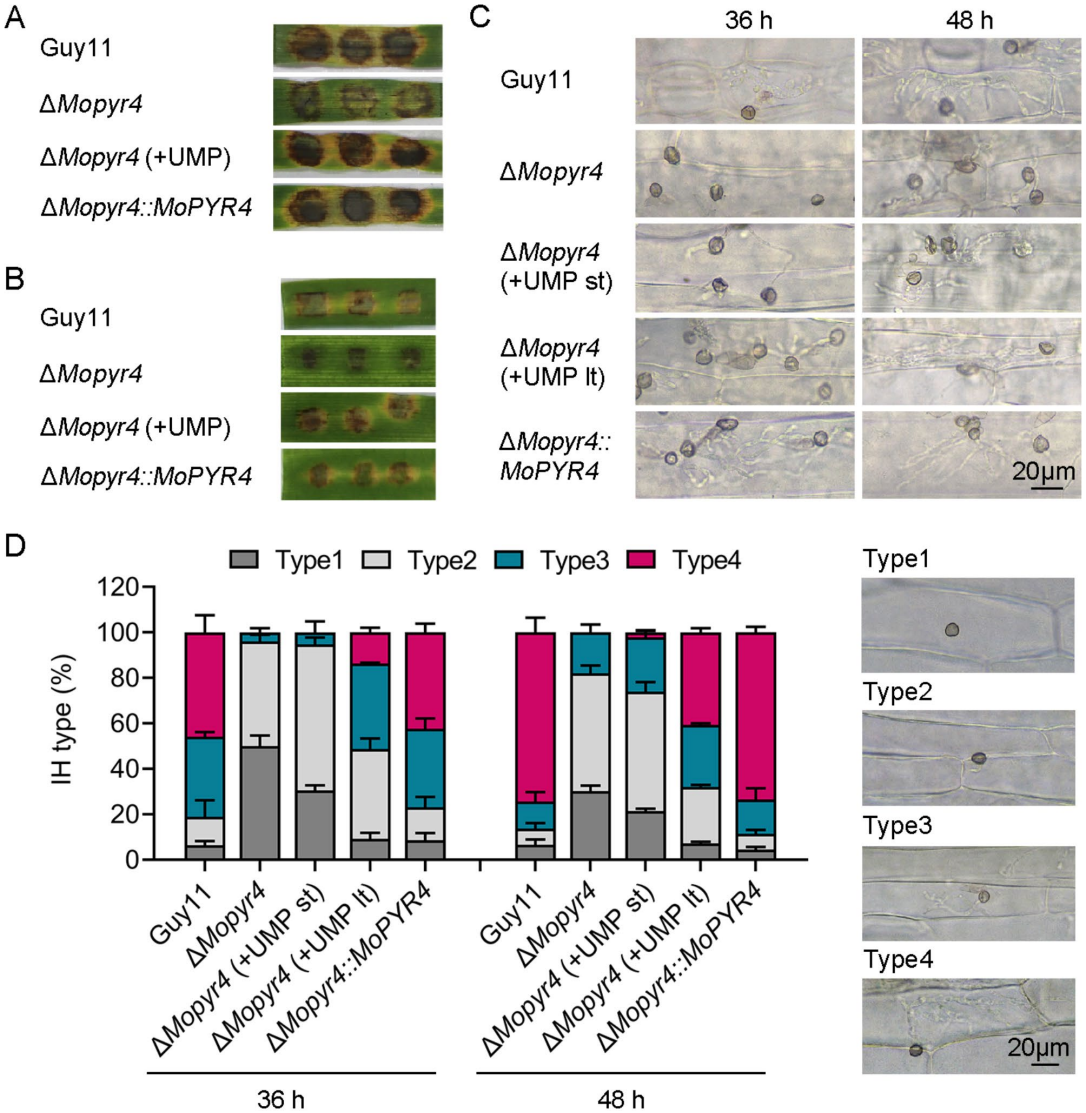
Restoration of pathogenicity and penetration in the Δ*Mopyr4* mutant after supplementation with exogenous UMP. (**A**) Disease spots on cut barley leaves inoculated with mycelial plugs of the Guy11, Δ*Mopyr4* mutant, complementary strain, and the Δ*Mopyr4* mutant supplemented with 5 mM exogenous UMP. (**B**) Disease symptoms on isolated barley leaves inoculated with conidial suspensions (20 µL, 5 × 10^4^ conidia/mL) of the Guy11, Δ*Mopyr4* mutant, complementary strain, and the Δ*Mopyr4* mutant supplemented with 5 mM exogenous UMP. (**C**) Penetration and growth of infectious hyphae in detached barley leaves inoculated with conidial suspensions (20 µL, 5 × 10^4^ conidia/mL) of the Guy11, Δ*Mopyr4* mutant, complementary strain, and the Δ*Mopyr4* mutant supplemented with 5 mM exogenous UMP. ‘lt’ indicates long-term addition of UMP both to the CM plates and conidial suspensions. ‘st’ indicates short-term addition of UMP only to the conidial suspensions after washing from the CM plates without UMP. Scale bar, 20 μm. (**D**) Penetration assays on cut barley leaves were quantified and statistically analyzed. Scale bar, 20 μm

We cut mycelia plugs of the same size from 7-day-old colonies of Guy11, Δ*Mopyr4*::*MoPYR4*, and two types of the Δ*Mopyr4* mutants grown on CM plates and CM plates supplemented with exogenous UMP, and inoculated them on isolated barley leaves for 4 days. The Δ*Mopyr4* from the CM plate produced only weak disease spots, while the Δ*Mopyr4* from the CM + UMP plate led to severe brown lesions and leaf yellowing on the barley leaves, which was consistent with the findings for Guy11 and Δ*Mopyr4*::*MoPYR4*, suggesting that the pathogenicity of Δ*Mopyr4* was restored by the exogenously added UMP (Fig. 4A). Next, in addition to the normal CM plates, we collected the conidia of the Δ*Mopyr4* strain on CM plates supplemented with exogenous UMP and diluted them using ddH_2_O supplemented with UMP. The conidial suspensions were then inoculated on isolated barley leaves for 4 days. As predicted, the pathogenic ability of the mutant conidia recovered to the level of that of the wild-type and complemented strains after exogenous UMP supplementation (Fig. 4B).

Moreover, we observed the formation of penetration pegs and the expansion of invasive hyphae inside detached barley leaves at 36 and 48 h after conidial inoculation under a microscope. To distinguish the degree of influence of the exogenous UMP on the infection ability of the mutants, the exogenous UMP addition was divided into long-term supplement (lt) and short-term supplement (st) groups (lt, the Δ*Mopyr4* mutants were cultured on CM plates supplemented with exogenous UMP before the conidia were washed off, and the exogenous UMP was added to the conidial suspension before inoculation onto the leaves; st, the Δ*Mopyr4* mutants were cultured on CM plates without exogenous UMP, and the exogenous UMP was added to only the conidial suspension before inoculation). As shown in Fig. 4C and D, the process of invasive hyphal formation and expansion was divided into four types (type 1, no invasive hyphae formed; type 2, only a single invasive hypha was formed; type 3, the invasive hyphae formed two or three branches; and type 4, the invasive hyphae produced multiple branches and expanded to adjacent cells). More than 90% of the appressoria of the wild-type and complemented strains formed invasive hyphae at 36 h post-inoculation (hpi), and the invasive hyphae branched and expanded rapidly. In contrast, the infection process of the appressoria of the Δ*Mopyr4* strain was extremely slow. At 48 hpi, approximately 70% of the invasive hyphae of Guy11 and Δ*Mopyr4*::*MoPYR4* were type 4, less than 20% were type 3, and very few appressoria were type 1. However, in the Δ*Mopyr4* mutant, approximately 80% of the invasive hyphae were type 1 or type 2, and only a few invasive hyphae branched into type 3 or type 4, showing that the infection process was still slow and lagging. These results suggest that the defects in the formation and expansion of invasive hyphae mediated by the appressorium were the main reason for the reduced pathogenicity of the mutant. Moreover, the addition of exogenous UMP, especially the long-term supplement, had a recovery effect on the infection process of the Δ*Mopyr4* mutant (Fig. 4C and D).

In conclusion, the Δ*Mopyr4* mutant has defects in development of invasive hyphae during infection, and it can be partly restored by addition of exogenous UMP.

### The appressorium formation and appressorium turgor pressure were defective in the Δ*Mopyr4* mutant but were restored with exogenous UMP

The formation and internal turgor accumulation of the appressorium are the keys for *M. oryzae* to invade host plants. Therefore, the following experiments further explored the specific reasons for the decreased infection ability of the Δ*Mopyr4* mutant from these two aspects, and verified the recovery effects of exogenous UMP on the recovery of these defects.

**Fig. 5:**
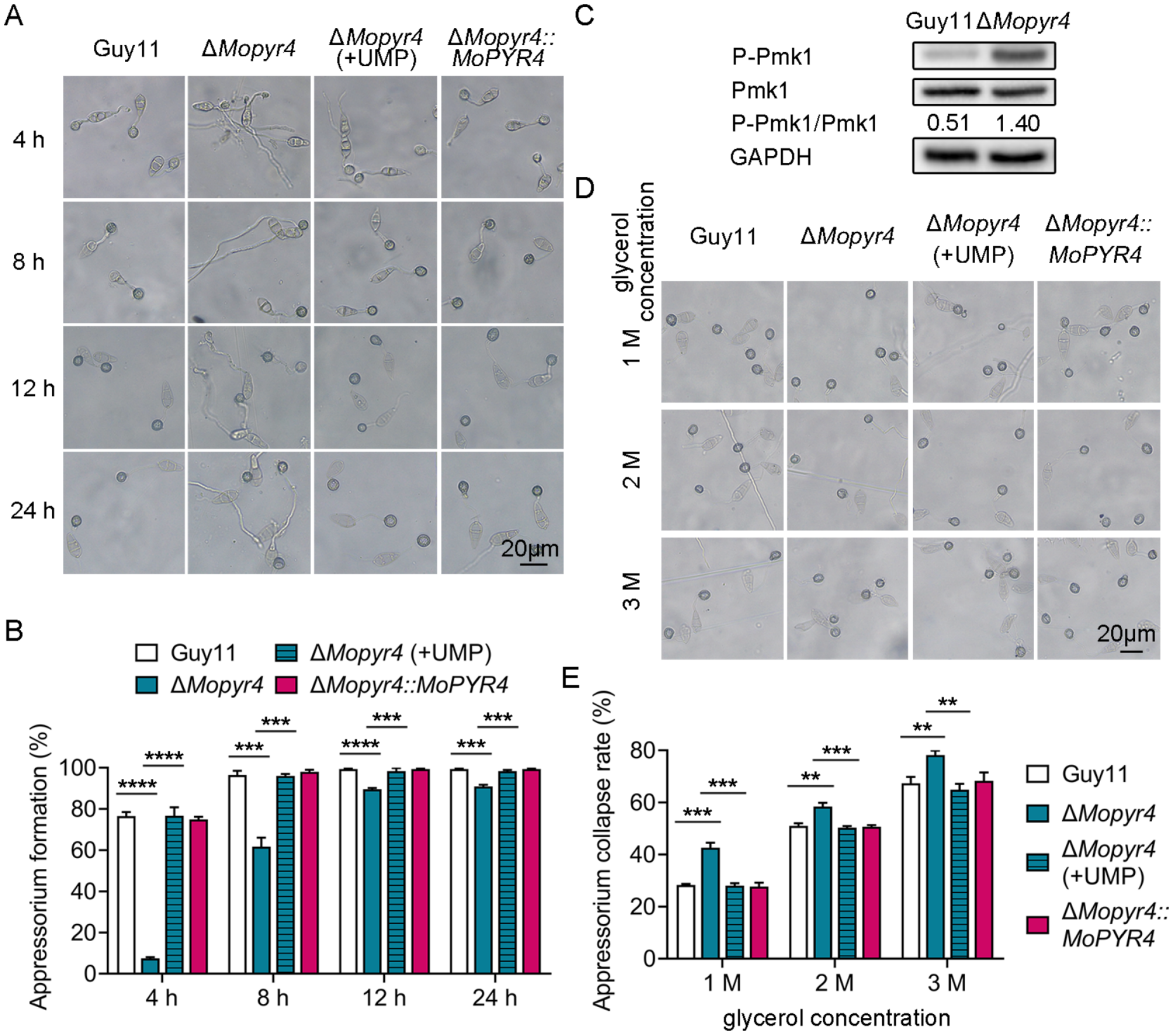
Restoration of appressorium formation and turgor pressure in the Δ*Mopyr4* mutant after supplementation with exogenous UMP. (**A**) Appressorium formation of the Guy11, mutant, mutant supplemented with 5 mM UMP, and complementary strains observed at different time points. Scale bar, 20 μm. (**B**) Statistical analysis of the appressorium formation rates of the Guy11, mutant, mutant supplemented with 5 mM UMP, and complementary strains at different time points. The data were calculated from three replicates. Asterisks indicate statistically significant differences (t-test, *** *P* < 0.001, **** *P* < 0.0001). (**C**) Immunoblotting of the phosphorylation level of Pmk1 in Guy11 and the Δ*Mopyr4* mutant. The phosphorylation level of Pmk1 was examined with an anti-P-Pmk1 antibody. The unphosphorylation level of Pmk1 was examined with an anti-Pmk1 antibody. The protein GAPDH was used as a loading control. The level of phosphorylated Pmk1 was calculated using the Formula P-Pmk1/Pmk1. (**D**) Appressorium collapse of the Guy11, mutant, mutant supplemented with 5 mM UMP, and complementary strains observed in 1 M, 2 M, or 3 M of glycerol solutions. Scale bar, 20 μm. (**E**) Statistical analysis of the appressorium collapse rates of the Guy11, mutant, mutant supplemented with 5 mM UMP, and complementary strains in 1 M, 2 M, or 3 M of glycerol solutions. The data were calculated from three replicates. Asterisks indicate statistically significant differences (t-test, ** *P* < 0.01, *** *P* < 0.001)

To induce appressorium development, we collected the conidia of Guy11, Δ*Mopyr4*::*MoPYR4*, and Δ*Mopyr4* grown on CM plates and Δ*Mopyr4* grown on CM + UMP plates and then inoculated them on artificial hydrophobic surfaces. The appressorium formation was observed at 4, 8, 12, and 24 hpi. The results showed that the appressorium formation rate of Δ*Mopyr4* was significantly lower than that of the wild-type and complemented strains at all time points, and at 24 hpi, almost all conidia of wild-type and complemented strains formed mature appressoria, while the appressorium formation rate of the mutant strain was less than 90% (Fig. 5A and B). Moreover, the lengths of the germ tubes of the *Mopyr4* mutant were much longer when most of the appressoria were formed, and the conidia that failed to form appressorium showed abnormal growth of the germ tube and inability to expand the tip (Fig. 5A). Therefore, there was a defect in the appressorium formation of Δ*Mopyr4*. In addition, the long-term exogenous addition of UMP effectively rescued the impairments in the appressorium formation of Δ*Mopyr4* (Fig. 5A and B).

According to the above results, the Δ*Mopyr4* mutant exhibited defects in appressorium formation, penetration peg formation, and invasive hyphal expansion (Figs. 4C and 5A and B, and 4D). Considering the regulation of these three infection-related processes by the Pmk1-MAPK signaling pathway [19], the phosphorylation levels of Pmk1 were detected in Guy11 and Δ*Mopyr4*. Figure 5C shows that the level of phosphorylated Pmk1 in the Δ*Mopyr4* strain was abnormally elevated, as determined by western blotting (WB), suggesting abnormal activation of the Pmk1-MAPK signaling pathway in the mutant strain.

Sufficient internal turgor pressure generated from glycerol accumulation in mature appressoria is a necessary condition for generating mechanical force to push the penetration peg through the host surface [17]. According to the excessive length of the germ tube of Δ*Mopyr4* during appressorium formation observed in Fig. 5A, we speculated that the transport efficiency of materials from conidia to the appressorium might be reduced, which could therefore affect turgor pressure accumulation inside the appressoria. Therefore, we performed an appressorium collapse experiment to measure the turgor pressure in the appressorium. As expected, the collapse rate of the appressoria of Δ*Mopyr4* was significantly greater than that of Guy11 and Δ*Mopyr4*::*MoPYR4* under different glycerol concentrations. The difference between the mutant and the wild-type strains was most significant under the 1 M glycerol treatment, as the collapse rate of the appressoria of Δ*Mopyr4* was approximately 14% greater than that of Guy11 (Fig. 5D and E). This result indicates that the internal turgor accumulation was impaired in the appressorium of Δ*Mopyr4*. In addition, the long-term exogenous addition of UMP to Δ*Mopyr4* reduced the collapse rate of the appressorium to the same level as that in Guy11 and Δ*Mopyr4*::*MoPYR4* (Fig. 5D and E), suggesting a recovery of the defect in turgor pressure accumulation in the appressoria of Δ*Mopyr4*.

Taken together, the Δ*Mopyr4* mutant has defects in appressorium formation and internal turgor pressure accumulation in the appressoria, and these can be restored by long-term addition of exogenous UMP.

### The degradation and transport of glycogen and lipid droplets were defective in the Δ*Mopyr4* mutant but were restored with exogenous UMP

**Fig. 6:**
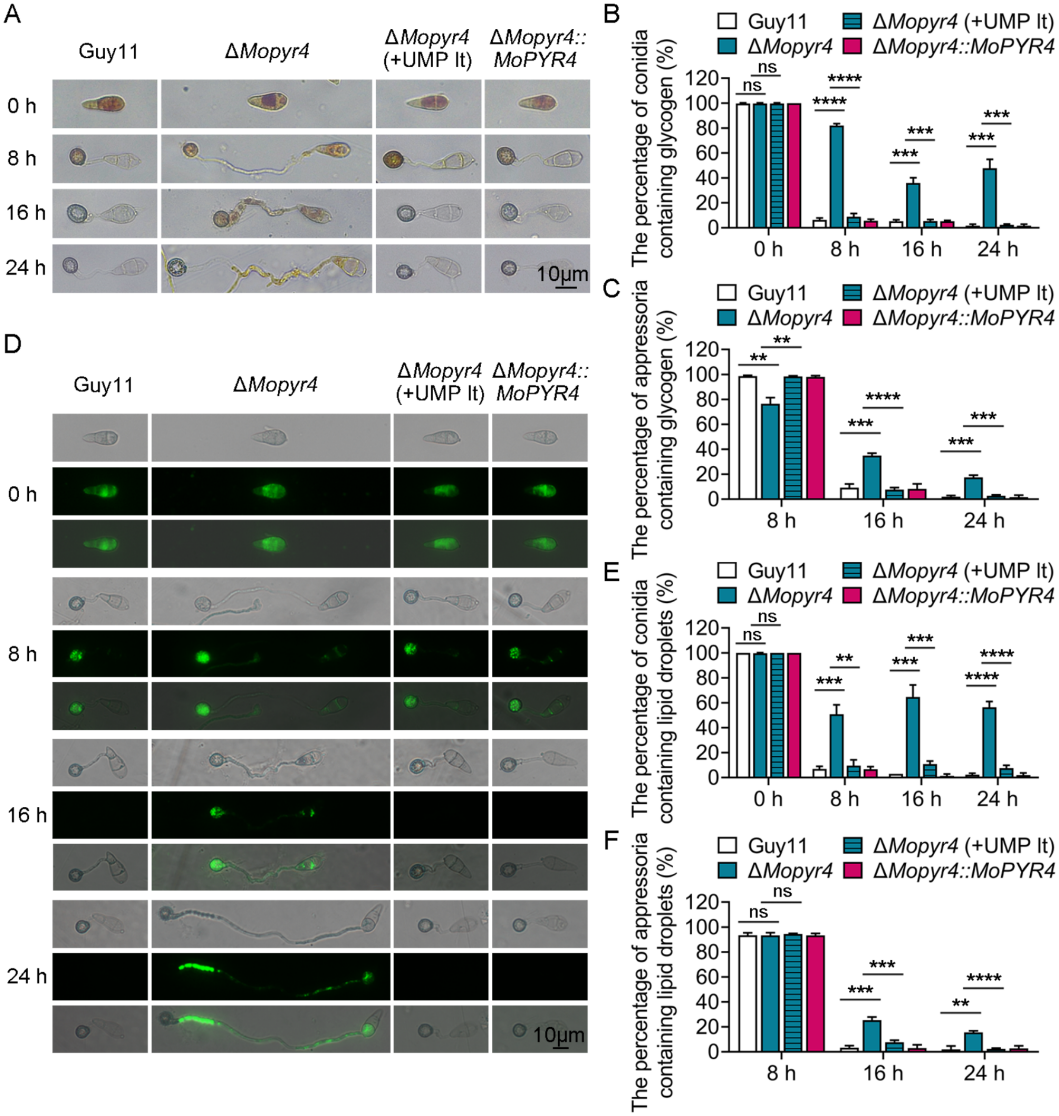
Restoration of glycogen and lipid droplet degradation and transport in Δ*Mopyr4* after the long-term addition of exogenous UMP. (**A**) Localization of glycogen in the conidia and appressoria of the Guy11, mutant, mutant supplemented with 5 mM UMP in the long term, and complementary strains at different time points during appressorium development. KI/I_2_ solution was used to stain glycogen at 0, 8, 16, and 24 hpi before observation. Scale bar, 10 μm. (**B**, **C**) Statistical analysis of the percentage of conidia (**B**)/appressoria (**C**) containing glycogen in the Guy11, Δ*Mopyr4*, Δ*Mopyr4*(+ UMP lt), and Δ*Mopyr4*::*MoPYR4* at different time points. The data were calculated from three replicates per treatment. At least 100 conidia/appressoria were counted per replicate. ‘ns’ indicates no statistically significant difference. Asterisks indicate statistically significant differences (t-test, ns *P* > 0.05, ** *P* < 0.01, *** *P* < 0.001, **** *P* < 0.0001). (**D**) Localization of lipid droplets in conidia and appressoria of the Guy11, mutant, mutant supplemented with 5 mM UMP in the long term, and complementary strains at different time points during appressorium development. BODIPY was used to stain lipid droplets at 0, 8, 16, and 24 hpi before observation under a fluorescence microscope. Scale bar, 10 μm. (**E**, **F**) Statistical analysis of the percentage of conidia (**E**)/appressoria (**F**) containing lipid droplets in Guy11, Δ*Mopyr4*, Δ*Mopyr4*(+ UMP lt), and Δ*Mopyr4*::*MoPYR4* at different time points. The data were calculated from three replicates per treatment. At least 100 conidia/appressoria were counted per replicate. ‘ns’ indicates no statistically significant difference. Asterisks indicate statistically significant differences (t-test, ns *P* > 0.05, ** *P* < 0.01, *** *P* < 0.001, **** *P* < 0.0001)

The accumulation of turgor pressure inside appressoria requires the autophagic degradation of glycogen and lipids and their successful transport from conidia to appressoria through germ tubes [31, 32]. Since the appressoria of the Δ*Mopyr4* mutant failed to accumulate sufficient internal turgor pressure (Fig. 5D and E), we continued to test whether there were impairments in the degradation and transport of glycogen and lipid droplets in the mutant. We induced the develoment of Guy11, Δ*Mopyr4*, and Δ*Mopyr4*::*MoPYR4* appressoria on transparent hydrophobic slides and then used KI/I_2_ solution and the fluorescent dye boron dipyrromethene (BODIPY) to stain glycogen and lipid droplets, respectively, before observation under a microscope at 0, 8, 16, and 24 hpi. Figure 6A and B show that glycogen biosynthesis in the conidia of the mutant did not differ from that in the conidia of the wild-type. However, the glycogen in the conidia of Δ*Mopyr4* was significantly more than that of Guy11, and the glycogen in the appressoria of Δ*Mopyr4* was significantly less than that of Guy11 at 8 hpi. When glycogen in most of the conidia and appressoria of Guy11 were both degraded at 16 and 24 hpi, the glycogen degradation rates in the conidia and appressoria of Δ*Mopyr4* were both significantly lower (Fig. 6A and B, and 6C). These results revealed obvious defects in the degradation and transport of glycogen by Δ*Mopyr4* during the appressorium development. As shown in Fig. 6D and E, and 6F, compared with those of Guy11, the degradation and transport of lipid droplets in Δ*Mopyr4* also tended to be impaired. Moreover, long-term supplementation with exogenous UMP effectively reversed the above defects in Δ*Mopyr4* (Fig. 6).

Taken together, there are delayed degradation and transport of glycogen and lipid droplets in the Δ*Mopyr4* mutant, and these could be rescued by long-term addition of exogenous UMP.

### MoPyr4 responds to oxidative stress and colocalizes with peroxisomes

**Fig. 7:**
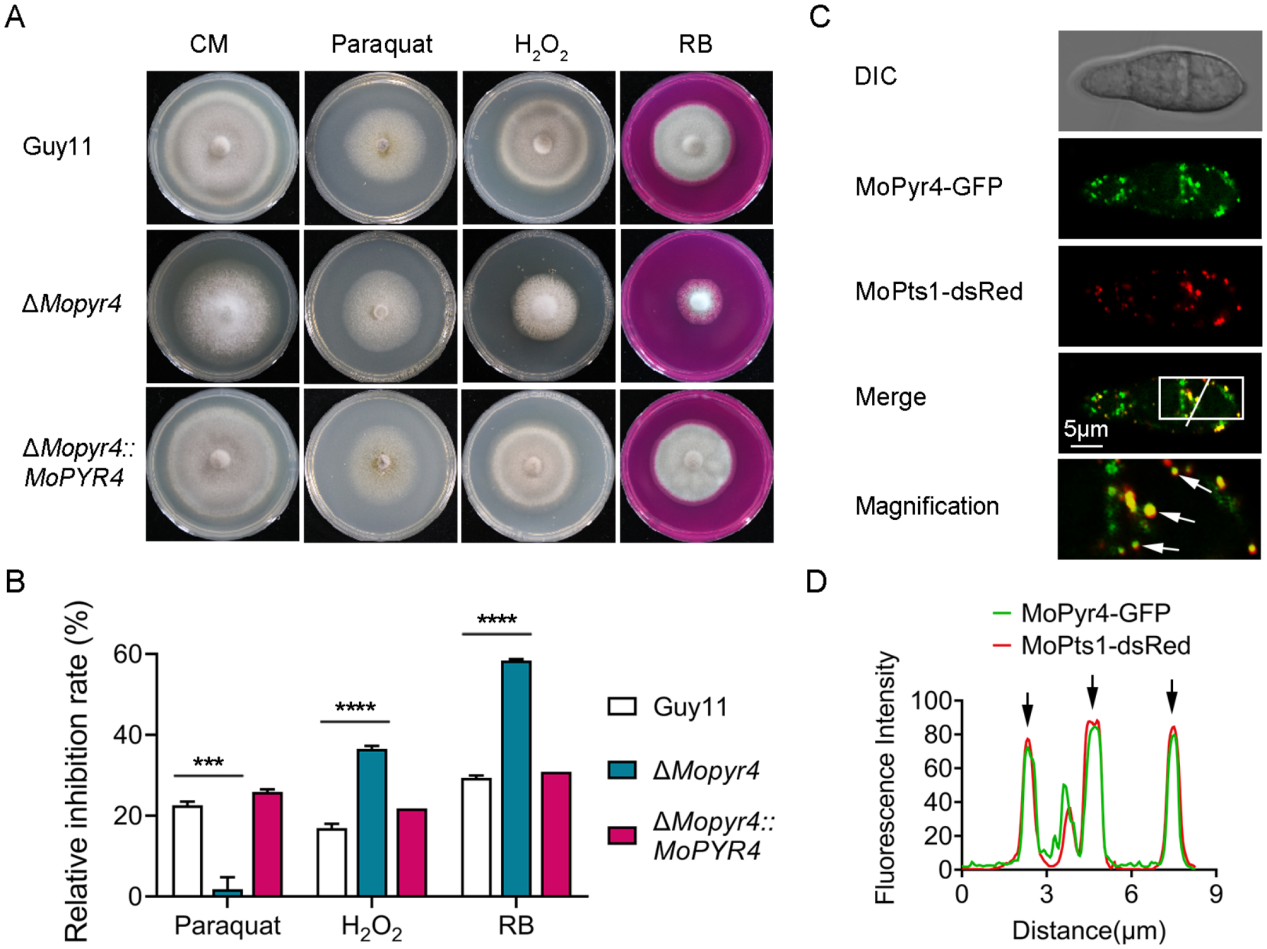
Hyphal growth of the Δ*Mopyr4* mutant on CM supplemented with oxidative stress factors and co-localization of MoPyr4 with peroxisomes. (**A**) Hyphal growth of the Guy11, mutant, and complementary strains on CM plates supplemented with 1 mM paraquat, 5 mM H_2_O_2_ and 50 µM RB. (**B**) Statistical analysis of relative growth inhibition rates of the Guy11, mutant, and complementary strains on CM plates supplemented with oxidative stress factors. The data were calculated from three replicates. Asterisks indicate statistically significant differences (t-test, *** *P* < 0.001, **** *P* < 0.0001). (**C**) Partly co-localization of MoPyr4-GFP with MoPts1-dsRed. Confocal fluorescence microscopy images (Zeiss LSM880, 63 × oil) of co-expressed dsRed-tagged protein and GFP-labeled protein were acquired. The overlapping fluorescence signals of GFP and dsRed in the merged image are framed with white borders and magnified in the magnified image, with white arrows denoting co-localization. Scale bar, 5 μm. (**D**) Line-scan graph showing the fluorescence intensities of green and red fluorescence signals, with black arrows denoting co-localized areas

Reactive oxygen species (ROS) burst is an important strategy in plant immunity that not only directly inhibits the growth of pathogens but also serves as an important signal to trigger other immune responses. Therefore, pathogens need to scavenge ROS to ensure the growth and expansion of invasive hyphae in plants [33, 34]. Figure 4C and D show the lower infection rate of Δ*Mopyr4* in host plants and the defects in the expansion of invasive hyphae to adjacent cells (type 4). Therefore, we considered the possibility of defects in the ROS clearance ability of Δ*Mopyr4* during infection. As a result, we explored the sensitivity of the mutants to oxidative stress. We added paraquat (1 mM), hydrogen peroxide (H_2_O_2_, 5 mM), and rose bengal (RB, 50 µM) to CM plates and measured the inhibitory effects of these oxidative stress factors on the wild-type, mutant, and complemented strains. As shown in Fig. 7A and B, the relative inhibition rates of Δ*Mopyr4* on the H_2_O_2_- and RB-supplemented plates were significantly greater than those of Guy11 and Δ*Mopyr4*::*MoPYR4*, indicating that the mutant was more sensitive to H_2_O_2_ and RB. Interestingly, Δ*Mopyr4* was significantly less sensitive to paraquat, and the agent had almost no inhibitory effect on the mutant, which was reversed for the other two agents. Taken together, these results indicate that the deletion of the *MoPYR4* gene results in significant changes in the susceptibility of *M. oryzae* to various oxidative stresses.

Several reports have shown that one of the major metabolic functions of peroxisomes is ROS scavenging, which is very important for fungal infection [33–35]. To investigate the relationship between MoPyr4 and peroxisomes, we observed the conidial distribution of MoPyr4-GFP and MoPts1-dsRed-labeled peroxisomes in *M. oryzae* via confocal fluorescence microscopy. The fluorescence microscope showed that MoPyr4 and MoPts1 were both scattered throughout the cytoplasm of the conidia (Fig. 7C). Although there was no complete co-localization, a certain number of obvious co-localization sites between MoPyr4 and MoPts1 were found, showing a partial co-localization pattern and implying a close relationship between them. We amplified and pointed out the regions of overlapping fluorescence signals and made a line-scan graph representing the fluorescence intensity, as shown in Fig. 7C and D. Overall, MoPyr4 partly colocalizes with peroxisomes in *M. oryzae*.

### MoPyr4 regulates the phosphorylation of Osm1 in response to hyperosmotic stress

**Fig. 8:**
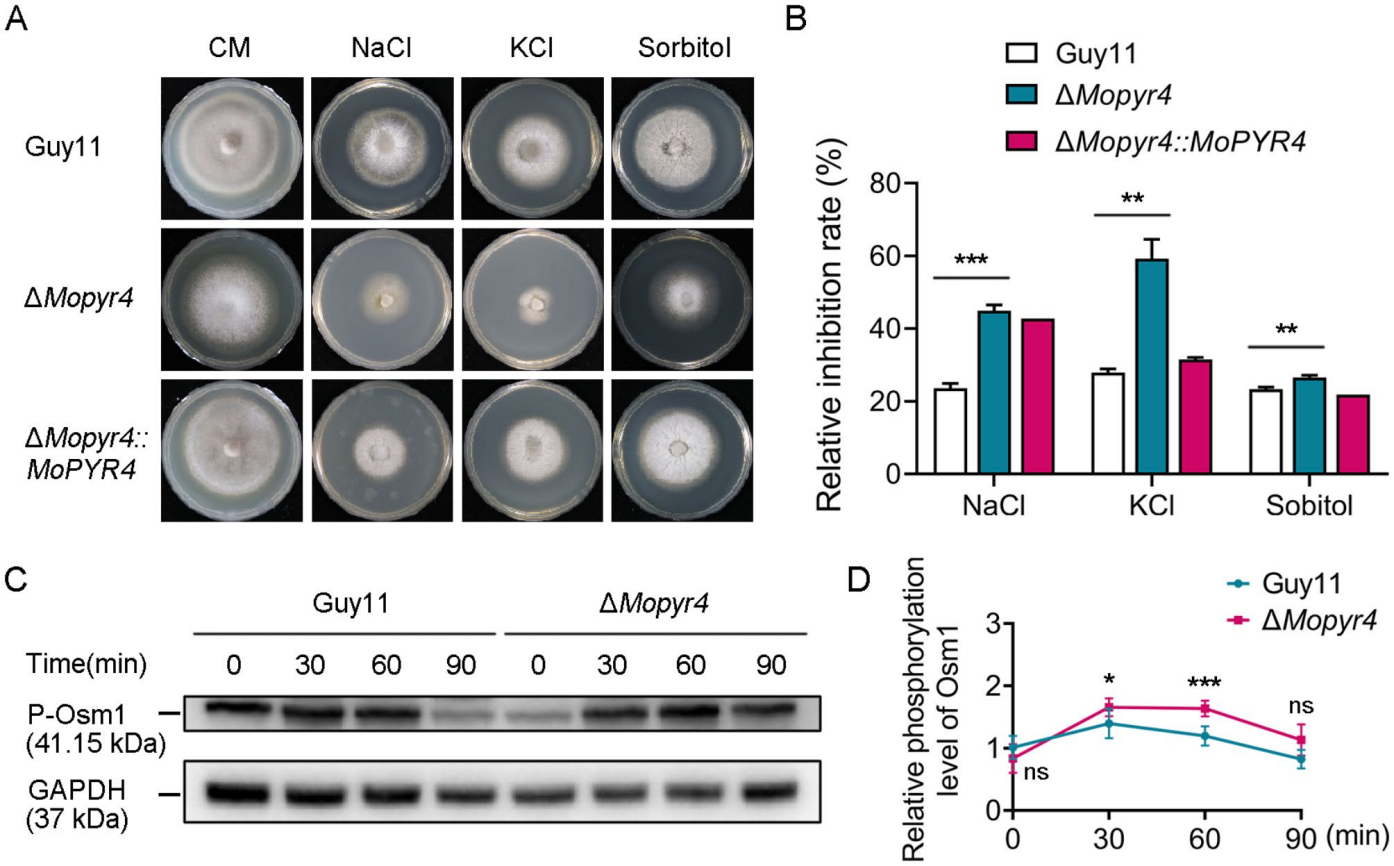
Hyphal growth of the Δ*Mopyr4* mutant strain on CM supplemented with hyperosmotic stress factors and the phosphorylation level of Osm1 in the Δ*Mopyr4* mutant. (**A**) Hyphal growth of the Guy11, mutant, and complementary strains on CM plates supplemented with 0.6 M NaCl, 0.6 M KCl, and 1 M sorbitol. (**B**) Statistical analysis of the relative growth inhibition rates of the Guy11, mutant, and complementary strains on CM plates with hyperosmotic stress factors. The data were calculated from three replicates. Asterisks indicate statistically significant differences (t-test, ** *P* < 0.01, *** *P* < 0.001). (**C**) Immunoblotting of the phosphorylation level of Osm1 in the Guy11 and mutant strains. The vegetative hyphae of the Guy11 and mutant strains were grown in liquid CM supplemented with 0.6 M NaCl for 0, 30, 60, or 90 min for hyperosmotic stress induction. The phosphorylation level of Osm1 was examined with an anti-P-Osm1 antibody. The protein GAPDH was used as a loading control. (**D**) Changes in the relative phosphorylation level of Osm1 in Guy11 and the mutant over time. The relative level of phosphorylated Osm1 was calculated using the following formula: P-Osm1/GAPDH. The data were calculated from three replicates. ‘ns’ indicates no statistically significant difference. Asterisks indicate statistically significant differences (t-test, ns *P* > 0.05, * *P* < 0.1, *** *P* < 0.001)

In addition to oxidative stress, the accumulation of degradation products caused by plant cell death during pathogen invasion exposes fungi to an environment with increased osmotic pressure, so it is also critical for pathogenic fungi to recognize and respond to external hypertonic environments [36]. Therefore, we investigated the sensitivity of the mutant to hypertonic stress. We calculated the relative inhibition rates of the hypertonic stress factors sodium ion (NaCl, 0.6 M), potassium ion (KCl, 0.6 M), and sorbitol (1 M) on Guy11, Δ*Mopyr4*, and Δ*Mopyr4*::*MoPYR4*, and found that the Δ*Mopyr4* mutant was significantly more sensitive to high concentrations of all three hypertonic stress factors (Fig. 8A and B). These results indicate that MoPyr4 plays an important role in the adaptability of *M. oryzae* to hypertonic stress.

The Osm1-MAPK signaling pathway in *M. oryzae* is homologous to the Hog1-MAPK signaling pathway in yeast, mediating the fungal response to hypertonic stress [37, 38]. We hypothesized that MoPyr4 is involved in the response of *M. oryzae* to hypertonic stress by regulating this pathway. Therefore, we detected differences in the phosphorylation levels of Osm1 in the mutant and wild-type strains under hypertonic conditions. The WB results showed that the uninduced vegetative hyphae of Guy11 maintained the phosphorylation of Osm1 at a relatively low level, and once transferred to media supplemented with 0.6 M NaCl to induce a hypertonic stress reaction, the phosphorylation level of Osm1 increased significantly within 30 min, and then decreased gradually within 1 h (Fig. 8C and D). There was no significant difference in the Osm1 phosphorylation level between Δ*Mopyr4* and Guy11 when the strains were not induced, but once induced by 0.6 M NaCl, the Osm1 phosphorylation level of Δ*Mopyr4* increased rapidly with increasing amplitude greater than that of Guy11 in the first 30 min and decreased slowly from 30 to 60 min. As a result, the phosphorylation levels of Osm1 in the mutant were significantly greater than those in the wild-type at both 30 and 60 min, suggesting that the Osm1-MAPK signaling pathway in the *MoPYR4* deletion mutant was abnormally active under hypertonic stress (Fig. 8C and D). These results show that MoPyr4 regulates the adaptability of *M. oryzae* to hypertonic stress and the relative phosphorylation level of Osm1.

### MoPyr4 interacts and partly co-localizes with MoAtg5

**Fig. 9:**
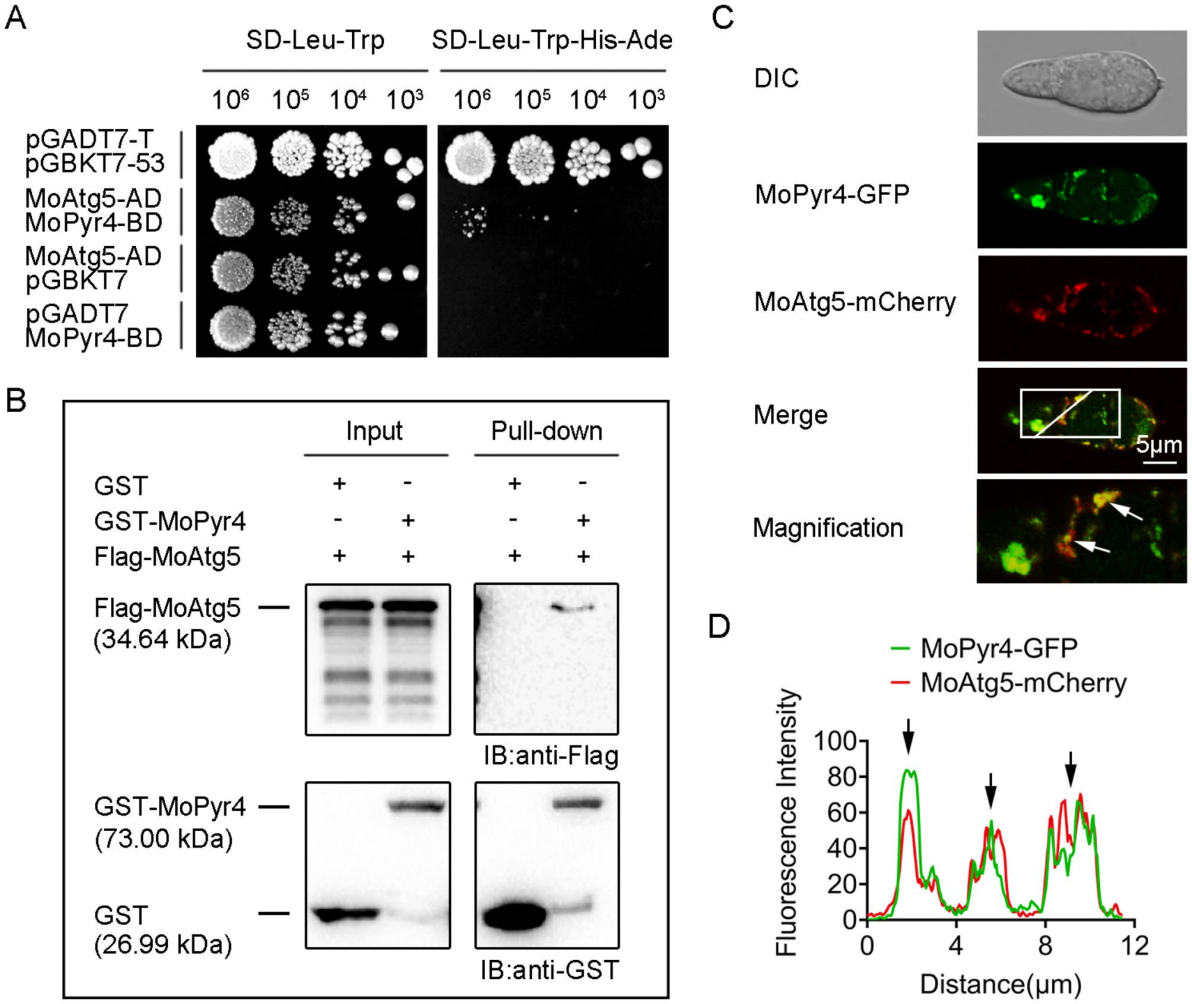
The interaction and co-localization of MoPyr4 with MoAtg5. (**A**) Yeast two-hybrid assay for examining the interaction between MoPyr4 and MoAtg5. The pair of plasmids pGADT7-T and pGBKT7-53 were used as the positive control. (**B**) GST pull-down assay between MoPyr4 and MoAtg5. GST-MoPyr4 and Flag-MoAtg5 were expressed in vitro. The proteins were purified, incubated with GST beads, and eluted. GST-MoPyr4 was detected with an anti-GST antibody, and Flag-MoAtg5 was detected using an anti-Flag antibody via WB. (**C**) Partial co-localization of MoPyr4-GFP with MoAtg5-mCherry. Confocal fluorescence microscopy images (Zeiss LSM880, 63 × oil) of co-expressing MoAtg5-mCherry and GFP-labeled MoPyr4 were captured in conidia. The overlapping fluorescence signals of GFP and mCherry in the merged image are framed with white borders and magnified, with white arrows denoting co-localization. Scale bar, 5 μm. (**D**) Line-scan graph showing the fluorescence intensities of green and red fluorescence signals, with black arrows denoting co-localized areas

MoPyr4, encoded by the gene MGG_12634, was identified among the proteins enriched by immunoprecipitation of MoAtg5-GFP identified by mass spectrometry (MS) (Table S1), thus, the following experiments were performed to verify the association between MoPyr4 and MoAtg5, the core protein of the autophagy pathway. First, we performed a yeast two-hybrid assay and preliminarily tested the interaction between MoPyr4 and MoAtg5 (Fig. 9A). Next, although a co-immunoprecipitation (Co-IP) assay failed to verify the interaction in vivo, a GST pull-down experiment provided additional evidence for this interaction (Fig. 9B). To observe the close relationship between MoPyr4 and MoAtg5 more directly, we co-expressed the fusion expression cassettes MoPyr4-GFP and MoAtg5-mCherry in *M. oryzae* and observed the fluorescence signals in the conidia. As shown in the confocal fluorescence microscopy images, MoPyr4-GFP and MoAtg5-mCherry were dispersed in the conidial cytoplasm, and some punctate and clustered locations of MoAtg5 were highly correlated with those of MoPyr4. The merged image and the line-scan graph of fluorescence intensities displayed the partial co-localized sites of the two proteins (Fig. 9C and D). In summary, these results prove the interaction and partial co-localization between MoPyr4 and MoAtg5.

### MoPyr4 positively regulates autophagic degradation

**Fig. 10:**
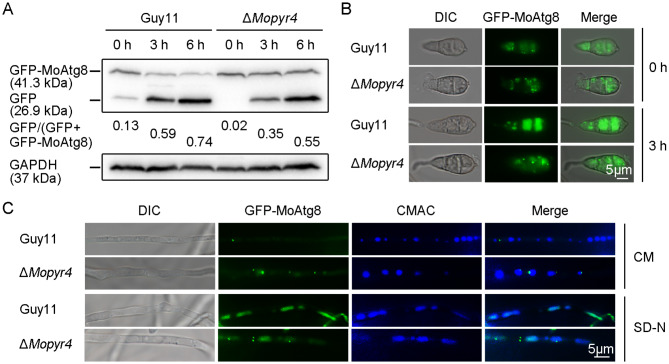
The degradation efficiency of autophagosomes was reduced in the Δ*Mopyr4* mutant. (**A**) Immunoblot analysis of GFP-MoAtg8 proteolysis in the Guy11 and mutant strains. The vegetative hyphae of the Guy11 and mutant strains expressing GFP-MoAtg8 were grown in liquid CM at 25 °C for 48 h and then transferred to liquid SD-N media for 0, 3, and 6 h for starvation induction. GFP and GFP-MoAtg8 were detected with an anti-GFP antibody in a WB assay. The degradation rates were calculated using the following formula: GFP/(GFP + GFP-MoAtg8). (**B**) Localization of GFP-MoAtg8 in the conidia of Guy11 and Δ*Mopyr4*. The conidia of the Guy11 and mutant strains were incubated in ddH_2_O for 0 and 3 h for starvation induction. The DIC and fluorescence images were examined under a fluorescence microscope. Scale bar, 5 μm. (**C**) Localization of GFP-MoAtg8 in the hyphae of Guy11 and Δ*Mopyr4*. The vegetative hyphae of the Guy11 and mutant strains expressing GFP-MoAtg8 were incubated in liquid CM and SD-N media, respectively. The vacuoles in vegetative hyphae were stained with 1 µM CMAC for 3 min. The DIC and fluorescence images were examined under a fluorescence microscope. Scale bar, 5 μm

To further explore how MoPyr4 regulates autophagy in *M. oryzae*, we examined the level of autophagic degradation in Δ*Mopyr4* and Guy11 under starvation conditions. During autophagic degradation, autophagosomes fuse with the vacuole, and the GFP-Atg8 labeled on the inner membranes of autophagosomes enters the vacuole, in which the Atg8 part of the fusion protein can be degraded by hydrolases, while the cleaved GFP is relatively resistant to hydrolysis and thus cannot be degraded. As a result, changes in the levels of free GFP and GFP-Atg8 are widely used to reflect intracellular autophagic affluxes, and GFP-Atg8 has become a well-used marker for autophagy activity detection [39, 40]. At present, the conserved function of GFP-Atg8 in *M. oryzae* has been confirmed [39, 40]. Therefore, the vegetative hyphae of the mutant and wild-type strains expressing GFP-MoAtg8 were precultured in liquid CM for 48 h and then transferred to liquid synthetic defined medium without amino acids and ammonium sulfate (SD-N) for starvation induction for 0, 3, or 6 h. First, we collected the vegetative hyphae at different time points to extract total protein for WB. After measuring the efficiency of free GFP cleavage from GFP-MoAtg8 in Guy11, we found that the ratio of GFP/(GFP + GFP-MoAtg8) was low before starvation induction, indicating a basic level of autophagy with low autophagy activity, while the ratio increased sharply during starvation induction, suggesting that GFP-MoAtg8 was degraded quickly and that a large amount of autophagic afflux was produced in response to the starvation induction. However, in Δ*Mopyr4*, the increase in the ratio of GFP/(GFP + GFP-MoAtg8) was very slow and limited under the nutrient deficiency conditions, and the efficiency of autophagy degradation was lower than that in the wild-type, indicating that the mutant had a lower level of autophagy activity in response to the starvation induction (Fig. 10A).

Then, we used fluorescence microscopy to observe the localization of GFP-MoAtg8 in the Δ*Mopyr4* and Guy11 strains. The fluorescence images of vegetative hyphae of Guy11 showed that GFP-MoAtg8 was mainly punctured outside vacuoles in the cytoplasm in the CM, implying that few autophagosomes entered the vacuoles for degradation. However, under nutrient deficiency conditions in SD-N media, the punctation of GFP-MoAtg8 was rare, and a large number of green fluorescence signals were dispersed and co-localized with the blue 7-amino-4-chloromethylcoumarin (CMAC)-stained vacuoles, indicating the occurrence of large autophagy affluxes and the efficient degradation of autophagosomes. However, in the hyphae of Δ*Mopyr4* incubated in the SD-N medium, in addition to the dispersed green fluorescence signal in the vacuole, we found many more punctured autophagosomes in the cytoplasm, indicating a slower autophagy process (Fig. 10C). Moreover, in the conidia of Δ*Mopyr4* treated with ddH_2_O for 3 h for starvation, many more GFP-MoAtg8 puncta were also observed, which was significantly different from that of Guy11 under the same conditions, where punctate GFP signals were rarely found (Fig. 10B). These fluorescence localization results are consistent with the WB immunoblotting results and validate our findings that MoPyr4 has a positive regulatory effect on starvation-induced autophagic degradation.

## Discussion

### The role of MoPyr4 in the growth and pathogenesis of *M. oryzae*

Pyrimidine nucleotides participate in a variety of metabolic pathways in cellular physiological and biochemical processes, including the synthesis of genetic material, the metabolism of nutrients, and the regulation of cell signals, therefore they are crucial to cellular life activities. In rice blast fungus, the only identified enzyme in the de novo pyrimidine nucleotide biosynthesis pathway is orotate phosphoribosyl transferase (OPRTase), which catalyzes the fifth enzymatic reaction step [41]. The study revealed that the absence of OPRTase leads to defects in the hyphal growth and pathogenicity of *M. oryzae* [41]. In this study, we knocked out the *MoPYR4* gene, which encodes the third enzyme (DHOase) in this pathway and systematically analyzed the abnormal phenotypes and defective pathogenicity of the Δ*Mopyr4* mutant, supplementing the understanding of the effects of pyrimidine nucleotide biosynthesis on fungal development and pathogenicity and further exploring the complexity of the regulatory effect of MoPyr4 on the growth and pathogenesis of *M. oryzae*.

Our study revealed that MM completely inhibited the growth of Δ*Mopyr4*, and this inhibition could be reversed by the addition of exogenous UMP (Figs. 1A and 3A and D), which suggested that the mutant was a nutrient-deficient strain lacking pyrimidine nucleotides and that MoPyr4 was required for pyrimidine nucleotide biosynthesis. This result is consistent with studies of OPRTase and reports on enzymes involved in the de novo purine nucleotide biosynthesis pathway in *M. oryzae* [41, 42]. In addition, although both the Δ*Mopyr4* and Δ*Mopyr5* strains grew slower than the wild-type strains on CM plates, the sparser and fluffier aerial hyphae of the Δ*Mopyr4* mutant and its reduced conidia-producing capacity were different from those of the Δ*Mopyr5* strain (Figs. 1 and 3) [41], indicating that different enzymes in de novo pyrimidine nucleotide biosynthesis pathway regulate fungal growth differently. We propose that pyrimidine nucleotide biosynthesis regulates fungal development through a sophisticated mechanism and that Mopyr4 may also regulate the growth of *M. oryzae* through a pathway in addition to the synthesis of UMP.

A report on *P. infestans*, the pathogen of potato blight, revealed that the relative expression of several enzymes in the de novo pyrimidine nucleotide biosynthesis pathway was upregulated in the early stage of biotroph infection and downregulated in the late stage of necrotrophic infection when the pathogenic fungus invaded the host diploid potato, suggesting the possibility of an important role for the de novo pyrimidine nucleotide biosynthesis pathway in the early infection of pathogenic fungi [9]. Moreover, the deletion of OPRTase in *M. oryzae* also reduced fungal virulence and inhibited the early infection of the mutant in the host plant [41]. Consistent with these results, our study revealed that the pathogenicity of Δ*Mopyr4* was weakened and that there was a significant delay in early infection of the Δ*Mopyr4* mutant in isolated barley leaves, which could be rescued by exogenous UMP addition (Figs. 2 and 4). Furthermore, UMP-related defects in appressorium formation, appressorium turgor pressure accumulation, and degradation and transport of glycogen and lipid droplets were also found in Δ*Mopyr4* (Figs. 5 and 6). These results comprehensively explain the impaired infection of the mutant, and reveal the positive role of MoPyr4-mediated pyrimidine nucleotide biosynthesis in the pathogenic process of *M. oryzae*.

However, the recovery of the infection process of Δ*Mopyr4* by exogenous UMP addition was far from what we expected. The short-term addition of exogenous UMP had a very limited effect on the infection process. The infection process pattern of Δ*Mopyr4* supplemented with UMP for short term were more similar to the mutant than the wild-type at both 36 and 48 hpi. Next, we found that the long-term exogenous addition of UMP obviously accelerated the infection process of Δ*Mopyr4*. This could be due to conidia containing more UMP or the compounds synthesized from UMP in long-term supplementation than in the short-term supplementation. However, the rate of infection of the mutant did not reach that of the wild-type and complemented strains, suggesting that long-term UMP supplementation still did not completely restore the infection process of the mutant (Fig. 4C and D). Therefore, in addition to the reduction in number, the conidia produced by Δ*Mopyr4* also had nutritional deficiencies that were difficult to recover. These results not only indicate that MoPyr4-mediated UMP biosynthesis is involved in the regulation of the formation and expansion of invasive hyphae but also reveal that the demand and utilization of UMP by *M. oryzae* during infection is long-term dependent. Acccording to the possible reasons why long-term supplementation of UMP could not completely compensate the phenotypes of the Δ*Mopyr4* mutant, we speculate that MoPyr4 might also participate in fungal pathogenicity and infection processes through some unknown functions in addition to UMP biosynthesis, and we also guess that the utilization efficiency of exogenous UMP is insufficient to supply rapid fungal infection.

### The participation of MoPyr4 in other pathways in *M. oryzae*

In the process of cell proliferation, large amounts of rRNA, DNA, and mRNA are synthesized to meet the needs of genome replication and transcription. When the demand for nucleotide synthesis in cells increases, cell proliferation will slow down or even stop if the supply of sufficient nucleotides cannot be met or the supply of nucleotide types is unbalanced [43]. Our study revealed that the growth of mycelia and conidiophores, appressorium formation, and the formation and expansion of invasive hyphae were inhibited in the Δ*Mopyr4* mutant, which may have first been caused by the slowing of cell proliferation.

Second, cells are constantly under metabolic pressure during cell proliferation, and autophagy is an important coping mechanism in addition to nutrient recycling. Under specific conditions, such as nutrient starvation conditions, autophagy is activated, during which double-membraned autophagosomes engulf cytoplasmic contents, organelles, and protein aggregates and transport them to vacuoles for degradation to satisfy the cell’s own nutrient supply and metabolic cycle and maintain the integrity and function of cells. Therefore, autophagy is also related to growth inhibition [22, 43]. Previous studies in *M. oryzae* have indeed shown that autophagy core gene-deleted mutants, such as Δ*Moatg5*, grow more slowly than wild-type strains when facing nutrient deficiency [44]. Some reports have also shown that the nutrient clearance function of autophagy is related to its role in maintaining cancer cell growth and tumor progression [45, 46]. In mouse lung cancer cells, autophagy is beneficial for maintaining the cellular nucleotide pool and energy balance to support cancer cell survival [45]. In addition, another study revealed that cancer cells that are rich in endogenous dNTPs or treated with dNTP precursors have a weaker response to rapamycin-induced autophagy, indicating that autophagy is negatively regulated by the size of the dNTP pool [47]. Therefore, it is speculated that the deletion of enzymes in the pyrimidine nucleotide synthesis pathway leads to an imbalance in the nucleotide pool in the cell, which may regulate the upstream level of autophagic degradation. Consistently, the reduced autophagy degradation in the pyrimidine nucleotide-deficient mutants in this study supports the view that the balance of the nucleotide pool is associated with the autophagy pathway, but the trend of positive regulation of autophagy by the pyrimidine nucleotide biosynthesis pathway is contrary to findings in mammalian cancer cells (Fig. 10). The Atg12-Atg5-Atg16 complex, in which Atg5 performs autophagy-related functions, is known to promote the lipidation of Atg8 during autophagosome formation [48, 49]. Although we verified the interaction of MoPyr4 with MoAtg5 in *M. oryzae* (Fig. 9), no significant difference was found in the level of MoAtg8-PE between the Δ*Mopyr4* mutant and Guy11 (data not shown). Therefore, the specific regulatory mechanism of MoPyr4 on the autophagy pathway and the function of the MoAtg5-MoPyr4 interaction still need to be further studied. We also observed that cytoplasmic MoPyr4 partially colocalized with peroxisomes in addition to the autophagy core protein MoAtg5 (Figs. 7C and D and 9C, and 9D), and MoPyr4 was involved in the regulation of fungal susceptibility to oxidative stress (Fig. 7A and B). These results suggest the possible participation of MoPyr4 in pexophagy.

The Pmk1-MAPK signaling pathway of *M. oryzae* not only regulates appressorium formation, but is also essential for the ability of the appressorium to penetrate the host plant and for the expansion of invasive hyphae in the host plant [20, 21]. In the Δ*Mopyr4* mutant, defects in the development and infection ability of appressoria were accompanied by an increase in the relative phosphorylation level of Pmk1 (Fig. 5C), indicating abnormal activation of the Pmk1-MAPK signaling pathway, and suggesting that MoPyr4 may be involved in fungal pathogenic mechanisms by maintaining the homeostasis of the Pmk1-MAPK signaling pathway, thereby contributing to appressorium formation, appressorium penetration, and invasive growth of *M. oryzae*. In addition, MoPyr4 participated in the Osm1-MAPK signaling pathway by regulating the relative phosphorylation level of Osm1, thereby regulating the adaptability of *M. oryzae* to hypertonic stress (Fig. 8), which also suggests a link between the Osm1-MAPK signaling pathway and MoPyr4. Moreover, Mps1-MAPK pathway is reported to participate in cell wall integrity, appressorium penetration, and invasive growth [19]. Long-term supplementation of UMP could not completely restore the number of invasive hyphae of type 3 and type 4 (Fig. 4C and D). We tested the phosphorylation level of Mps1 in Guy11 and the Δ*Mopyr4* mutant by WB, and found the increased phosphorylation levels of Mps1 in the Δ*Mopyr4* mutant, suggesting abnornal activation of the Mps1-MAPK signaling pathway and implying the perturbation of cell wall integrity (Figure S3). Therefore, the roles of MoPyr4 in cell wall integrity are also worth to be further studied.

**Fig. 11:**
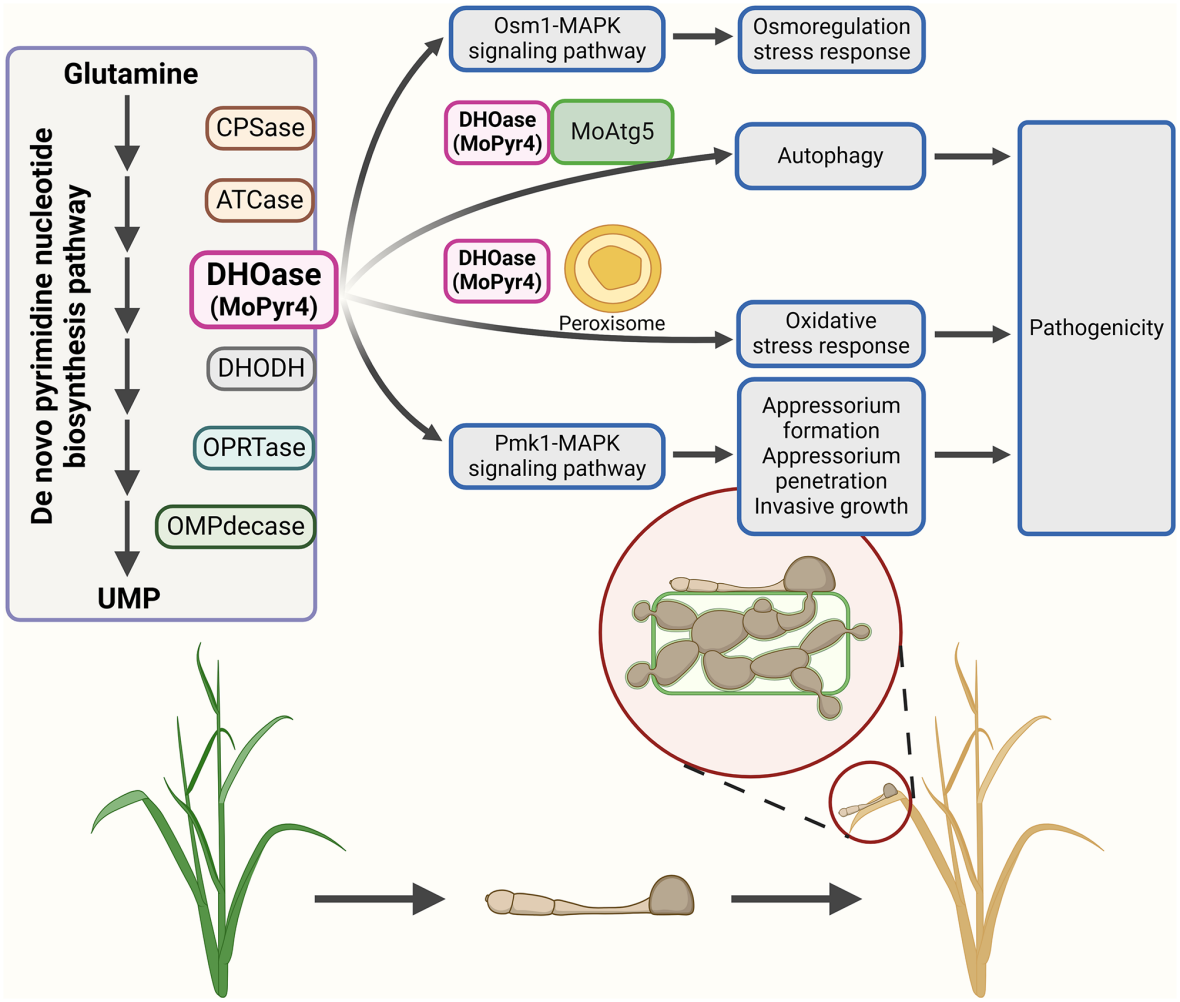
Model of MoPyr4 from the de novo pyrimidine nucleotide biosynthesis pathway affecting pathogenicity by regulating autophagy and other pathways in *M. oryzae*

Based on our findings on the role of MoPyr4 in the autophagy pathway, peroxisome-mediated oxidative stress response, Pmk1-MAPK signaling pathway, and Osm1-MAPK signaling pathway-mediated hyperosmotic stress response, we propose a model in which MoPyr4 co-regulates autophagy and other pathways to affect pathogenicity in *M. oryzae*. MoPyr4 from the de novo pyrimidine nucleotide biosynthesis pathway not only interacts with MoAtg5 to positively regulate autophagy in *M. oryzae*, but also regulates the fungal response to oxidative stress through co-localization with peroxisomes, and it is required for the development of appressoria and the growth of invasive hyphae by participating in the regulation of the Pmk1-MAPK signaling pathway. Therefore, MoPyr4 is essential for the virulence of *M. oryzae* through crosstalk with these three pathways. In addition, MoPyr4 is also related to the fungal response to hyperosmotic stress through regulating the Osm1-MAPK signaling pathway, which is not directly involved in appressoria-mediated infection, but is crucial for the recognition and response of pathogenic fungi to hyperosmotic environments after penetration and invasion (Fig. 11).

In summary, this study identified MoPyr4 (DHOase), the third enzyme in the de novo pyrimidine nucleotide biosynthesis pathway in *M. oryzae*, and revealed its ability to regulate fungal growth and virulence through UMP biosynthesis. We also found that MoPyr4 is required for the external stress response and pathogenic mechanism of *M. oryzae* by co-regulating the autophagy pathway, oxidative stress response mechanism, Pmk1-MAPK signaling pathway, and Osm1-MAPK signaling pathway.

## Materials and methods

### Fungal strains and culture conditions

The wild-type strain of *M. oryzae* used in this study was Guy11. In the routine experiments, mycelia of the wild-type, mutant, and complemented strains were cultured on solid complete medium (CM) plates at 25 °C under a 16 h- light/8 h- dark photoperiod [50]. To verify that the mutant strain was nutritionally deficient, solid minimal medium (MM) plates were also used. For the oxidative stress experiments, 1 mM paraquat dichloride, 5 mM hydrogen peroxide (H_2_O_2_), and 50 µM rose bengal (RB) were added to the CM plates. For the hyperosmotic stress experiments, 0.6 M sodium chloride (NaCl), 0.6 M potassium chloride (KCl), and 1 M sorbitol were added to the CM plates, as hyperosmotic stress factors. For the stress experiments, the strains were cultured on the above plates at 25 °C in the dark. For protein, DNA, and RNA extraction, the mycelial plugs of the fungi were cut from the solid media, shattered by concussion, and then cultured in liquid CM in a shaker at 25 °C and 150 rpm for 48 h. For the starvation-induced autophagy, the hyphae precultured in liquid CM were transferred to liquid synthetic defined media without amino acids and ammonium sulfate (SD-N) for another 0, 3, and 6 h. In the experiments involving exogenous UMP supplementation, a final concentration of 5 M UMP was added to the solid medium plates or conidial suspensions.

### Target gene knockout and complementation

To obtain the *MoPYR4*-knockout mutants, we first cloned approximately 2.0 kb of upstream and downstream fragments of the target *MoPYR4* gene from the genomic DNA of Guy11 by PCR with a superfidelity enzyme using MGG_12634upF/R and MGG_12634downF/R as primers. We also amplified the hygromycin-resistance gene (*HPH*) fragments with the primers HPH-F/R by using pCB1003 as a template. Moreover, the plasmid DNA of the pKO3A knockout vector was digested by two restriction endonucleases, FastDigest HindIII and FastDigest XbaI [51]. The three cloned DNA fragments and the HindIII/XbaI-linearized vector were isolated and purified by DNA gel electrophoresis and a DNA purification kit, respectively. We connected them with 2 × Basic Assembly Mix and transferred the ligase linking products into *Escherichia coli* DH5α and *Agrobacterium* AGL1 successively. Then, we introduced AGL carrying knockout vectors into the wild-type Guy11 so that the target *MoPYR4* gene could be replaced by *HPH* in the Guy11 genome via *Agrobacterium tumefaciens*-mediated transformation (ATMT) according to the principle of homologous substitution. We screened the transformants on CM plates supplemented with hygromycin, extracted the genomic DNA of the candidate transformants, and used PCR and quantitative real-time PCR (qRT-PCR) to confirm the successful knockout of the target gene. The qRT-PCR was performed by using TB Green^®^ Premix EX Taq™ as previously described [52, 53]. Finally, the Δ*Mopyr4* mutants were obtained.

To obtain the complemented strain Δ*Mopyr4*::*MoPYR4*, we cloned the *MoPYR4* sequence containing 2 kb of its native promoter via PCR as previously described, and fused it to the EcoRI/SmaI-digested pKD5 (containing GFP) vector. ATMT was used to insert the fusion cassette into the Δ*Mopyr4* mutant. The transformants were screened and verified by fluorescence observation. After confirming the recovery of phenotypes, the complemented strain Δ*Mopyr4*::*MoPYR4* was obtained. The PCR primers used are listed in Table S2.

### Phenotypic analyses

For vegetative growth examination, we cut the same-sized mycelial plugs from the edge of the 7-day-old colonies of each strain and inoculated them on the center of each plate of the corresponding medium. Each treatment was repeated three times. The plates were cultured at 25 °C under a 16 h- light/8 h- dark photoperiod. Then, we measured the colony diameters and took photos of the colonies at 7 dpi. For the conidiation measurements, we added 3 mL of ddH_2_O to each 7-day-old colony and collected all the conidia by centrifugation. We removed all the supernatants and added 3 mL of ddH_2_O to each centrifuge tube. Then, we counted and calculated the concentration of conidia in each conidial suspension by using a hemocytometer under a microscope. To induce the formation of appressoria, we collected and washed the conidia of each strain with ddH_2_O and diluted them to a conidial suspension of 5 × 10^4^/mL. The conidial suspensions were inoculated on transparent hydrophobic slides at 20 µL per drop, placed in a humidifying box, and incubated at 22 °C in the dark. We observed and calculated the appressorium formation rate at 4, 8, 12, and 24 hpi by using a microscope. To assess the internal appressorium turgor, the collapse rate of the appressoria was calculated in 1, 2, and 3 M glycerol solutions as previously described [31]. To examine the degradation and transport of glycogen and lipid droplets, KI/I_2_ solution and the fluorescent dye boron dipyrromethene (BODIPY) were used to stain glycogen and lipid droplets in both conidia and appressoria at 0, 8, 16, and 24 hpi. We observed and calculated the results by using a microscope. The above experiments were performed with three replicates per treatment, and at least 100 conidia/appressoria were counted per replicate.

### Pathogenicity and infection assays

In the pathogenicity assays on the barley, we cut 6 cm long leaves from 7-day-old potted-planted barley (*Hordeum vulgare* cv. ZJ-8). To observe the pathogenicity of the mycelia, we cut the same-sized mycelial plugs from the edge of the colonies and inoculated them on detached barley leaves at 25 °C for 4 d. Each leaf was inoculated with three mycelial plugs, and each strain was examined on three leaves. To observe the pathogenicity of the conidia, we collected conidia from 7-day-old colonies, washed them with ddH_2_O three times, and diluted the suspensions to a concentration of 5 × 10^4^/mL by counting them with a hemocytometer. The conidial suspensions of each strain were inoculated on three barley leaves at 20 µL per droplet, and three droplets per leaf at 25 °C for 4 d before recording. In the infection assays, the parts of the barley leaves inoculated with conidial droplets were cut at 36 and 48 hpi, and soaked in methanol until they were completely decolorized, and then fixed with an alcoholic lactophenol solution. We observed and analyzed the type of appressorium-mediated penetration and the expansion of invasive hyphae in fixed leaves under a microscope [54]. For the virulence test on rice, the susceptible *Oryza sativa* cv. CO-39 was chosen and cultured in pots for 2 weeks. The conidial suspensions (5 × 10^4^/mL) obtained from the 7-day-old colonies were diluted with 0.2% gelatin solution and sprayed on the rice seedlings at 2 mL per pot, with 3 pots for each strain. The rice seedlings were cultured at 22 °C in the dark for the first 2 d and then at 25 °C under a 16 h- light/8 h- dark photoperiod for another 5 d. Then, the severity of the disease in the rice of each strain was observed and photographed.

### Fluorescence observations

To observe the co-localization of MoPyr4 with the other two proteins, we constructed two fusion expression cassettes, MoAtg5-mCherry and MoPts1-dsRed, by using the pKD3-mCherry and pKD3-dsRed vectors, respectively, through the same method as that used for MoPyr4-GFP. MoPts1-dsRed was used to label peroxisomes [55, 56]. Then, they were transferred into the complemented strain Δ*Mopyr4*::*MoPYR4* by ATMT so that MoPyr4-GFP could be co-expressed with MoAtg5-mCherry or MoPts1-dsRed, and fluorescence co-localization could be observed. We captured images of two-track fluorescence signals with ZEN software by using an LSM880 laser scanning confocal microscope under a 63×/1.4 oil objective. The wavelength of the green fluorescent signal (ILEXTrack1) was 488 nm, and the wavelength of the red fluorescent signal (ILEXTrack2) was 561 nm. The fluorescence intensities of the fluorescence signals were analyzed by ImageJ software and are presented in the line-scan graphs.

To observe the localization of the autophagosomes, we constructed GFP-MoAtg8 with a native promoter by using the pKD3-GFP vector and then introduced it into the wild-type and mutant strains by ATMT. After the vacuoles were stained with 1 µM Cell Tracker Blue 7-amino-4-chloromethylcoumarin (CMAC) for 3 min, we observed green and blue fluorescence signals simultaneously under a fluorescence microscope. The primers used are listed in Table S2.

### Western blot (WB) analyses

To obtain the proteins used for WB, the hyphae of Guy11 and Δ*Mopyr4* were cultured in liquid CM in a shaker at 25 °C and 150 rpm for 48 h. For hyperosmotic stress induction, the precultured hyphae were divided into four equal parts and transferred to liquid CM supplemented with 0.6 M NaCl for another 0, 30, 60, or 90 min before collection. For starvation-induced autophagy, the precultured hyphae were transferred to liquid SD-N for another 0, 3, or 6 h. The hyphae were collected and then ground in liquid nitrogen to extract the total proteins. To detect the phosphorylation level of Pmk1, the TCA-acetone method was used to isolate proteins. We measured the protein concentrations with an enhanced BCA protein assay kit, and generated a concentrated gel and a separation gel for polyacrylamide gel electrophoresis (PAGE) with a rapid PAGE gel preparation kit. For immunoblotting Pmk1, we used an anti-phosphorylated Pmk1 antibody and an anti-non-phosphorylated Pmk1 antibody. For the WB analysis of Osm1 phosphorylation, an anti-phosphorylated Osm1 antibody was used. To measure autophagy afflux, we probed GFP and GFP-MoAtg8 with an anti-GFP antibody. In this study, the anti-GAPDH antibody was used as an internal control, and the secondary antibodies used were goat anti-rabbit/mouse IgG HRP. In the last step, the PVDF membrane was incubated with an enhanced chemiluminescence HRP kit for 3 min, after which the results of protein immunoblotting were detected by a gel imaging analyzer. The amounts of protein bands were measured by densitometric analysis with ImageJ software.

### Yeast two-hybrid assays

To detect the MoPyr4-MoAtg5 interaction in the yeast two-hybrid system, we cloned the coding sequence (CDS) fragment in the cDNA of *MoATG5* into the prey vector pGADT7 (AD) with the primers listed in Table S2, and the CDS fragment of *MoPYR4* was amplified and fused into the bait vector pGBKT7 (BD). Another pair of vectors of confirmed interaction proteins, pGADT7-T and pGBKT7-53, was used as the positive control. According to the instructions of the manufacturer of Matchmaker gal4 two-hybrid system 3, we co-transformed those pairs of vectors into the yeast strain Y_2_H Gold and observed the growth of each transformant on SD-Leu-Trp auxotrophic medium and SD-Leu-Trp-Ade-His synthesis medium at 30 °C at 5 dpi.

### GST pull-down assays

To confirm the interaction between MoPyr4 and MoAtg5, we first constructed GST-MoPyr4 and Flag-MoAtg5 fusion cassettes for GST pull-down assays. We cloned the CDSs of the target genes using cDNA as a template with the primers listed in Table S2 and fused them with pGEX-4T-1 (containing GST) and the pET21a vector (containing 3 × Flag). The ligase-linked products were transferred into the *E. coli* expression strain BL21. Then, we used 4 mM isopropyl-β-D-thiogalactoside (IPTG) to induce the expression of the target protein in BL21 cells at 18 °C for 16 h. After induction, whole-cell bacterial lysates were extracted by an ultrasonic cell disruptor, and the proteins were detected by SDS-PAGE with Coomassie blue staining. Subsequently, the bacterial lysates with GST-tagged protein were incubated with glutathione agarose beads at 4 °C for 3 h, and the bacterial lysates containing Flag-tagged protein were added and incubated at 4 °C for another 2 h. After incubation, we obtained the eluted proteins with elution buffer (10 mM GSH, 50 mM Tris, pH = 8.0) and detected the interactions via WB using an anti-GST antibody and an anti-Flag antibody.

### Statistical analyses

For the statistical significance analyses, unpaired two-tailed Student’s t-test were performed using GraphPad Prism 8 software for comparisons between two groups. Each result is shown as the mean ± standard deviation (s.d.) from three replicates. *P*-values were determined by bilateral t-tests; ns *P* > 0.05, * *P* < 0.05, ** *P* < 0.01, *** *P* < 0.001, **** *P* < 0.0001. ‘ns’ indicates no statistically significant difference, and asterisks indicate a statistically significant difference.

### Electronic supplementary material

Below is the link to the electronic supplementary material.

Supplementary Material 1

Supplementary Material 2

## Data Availability

The datasets used or analyzed during the current study are available in the manuscript and supplementary material.
