# Supplementary material for: Dihydroorotase MoPyr4 is required for development, pathogenicity, and autophagy in rice blast fungus
Source: Cell Commun Signal. 2024 Jul 15;22:362. doi: 10.1186/s12964-024-01741-4 (PMC11247805; doi:10.1186/s12964-024-01741-4)

**Supplementary materials**

**Table S1. The identification of MoPyr4 in MoAtg5-GFP mass spectrometry data.**

| Gene ID | MGG_12634 |
| --- | --- |
| Gene name | Dihydroorotase |
| Coverage | 4.69 |
| Peptides | 2 |
| PSMs | 2 |
| MW [kDa] | 46.0 |

**Table S2:**. **List of Primers in this study**

| Name Sequence (5’ - 3’) | |
| --- | --- |
| Primers used for gene knockout | |
| MGG_12634upF | GCCCGGGAGATGGGGGAGGCTAACTGACACTCTAGAAGCAAGCCTCTTGTCTTG |
| MGG_12634upR | CAAAATAGGCATTCATTGTTGACCTCCACTAGGCTTGACACGCAAGCAAGAC |
| MGG_12634downF | TCGTCCGAGGGCAAAGGAATAGAGTAGATGGCTGGCTTGTATGGGCTTGG |
| MGG_12634downR | TCACGACGTTGTAAAACGACGGCCAGTGCCAAGCTTAAGCCCACCACCAAGAAG |
| MGG_12634upyzF | CCGTATTACAATCCGAGTG |
| MGG_12634innerF | GGATTGGGACGGGTTGGGAAT |
| MGG_12634innerR | AGAACCTGAGCCACAGTGG |
| HPH-F | TAGTGGAGGTCAACAATGAATG |
| HPH-R | CATCTACTCTATTCCTTTGCCC |
| pKO1B-cxyzF | ACCACTACCAGCAGAACA |
| HPH-cxyzR | GGGCGAACTTAAGAAGGTATGA |
| HPH-cxyzF | GGACAATGGCCGCATAACA |
| pKO1B-cxyzR | AAACTGAAGGCGGGAAAC |
| HPH-cxR | GGGCGAACTTAAGAAGGTATGA |
| HPH-cxF | GGACAATGGCCGCATAACA |
| Tubulin1k-F | CCATCCCGAGCTTGTTGATA |
| Tubulin1k-R | GTAGTTCAGGTCACCGTATGAG |
| Primers used in fluorescent observation | |
| PYR4-GFP-F | TCACAATCACTAGTGAATTCTAGGTTCGGACGGTTGAG |
| PYR4-GFP-R | CCCTTGCTCACCATCCCGGGAACCCACTGCAGAGTGAAGA |
| ATG5-mCherry-F | CAATCACAATGGCCGGATCCATGGCTTCGCCGCGCCGATC |
| ATG5-mCherry-R | CCCTTGCTCACCATCCCGGGTAATGGCACGACGGTTAAAC |
| GFP-ATG8-F | CGAGCTGTACAAGTCTAGAATGCGCTCCAAGTTCAAGGAC |
| GFP-ATG8-R | TACTGCAGGTCGACTCTAGACTCGACTTCCTCAAACAGGTC |
| Primers used for yeast two-hybrid assay | |
| ATG5-AD-F | GGAGGCCAGTGAATTCATGGCTTCGCCGCGCCGATCAG |
| ATG5-AD-R | CGAGCTCGATGGATCCTAATGGCACGACGGTTAAAC |
| PYR4-BD-F | TGGCCATGGAGGCCGAATTCATGCTACATGATCTTATCGC |
| PYR4-BD-R | CGCTGCAGGTCGACGGATCCAACCCACTGCAGAGTGAAGA |
| Primers used for GST Pull-down | |
| ATG5-His-F | TGATGACGACAAGGTCGACATGGCTTCGCCGCGCCGATCAG |
| ATG5-His-R | CGAGTGCGGCCGCAAGCTTTAATGGCACGACGGTTAAAC |
| PYR4-GST-F | GCGTGGATCCCCGGAATTCATGCTACATGATCTTATCGC |
| PYR4-GST-R | CGAGTCGACCCGGGAATTCAACCCACTGCAGAGTGAAGA |

**Table S3. List of the sources of the materials mentioned in** **Materials and methods**

| Materials | Sources |
| --- | --- |
| Guy11 | Nicholas J. Talbot from the laboratory of the University of Exeter, UK |
| paraquat dichloride(H_2_O_2_) | Macklin, China |
| hydrogen peroxide | Sinopharm Chemical Reagent, China |
| sodium chloride (NaCl) | Sinopharm Chemical Reagent, China |
| potassium chloride (KCl) | Sinopharm Chemical Reagent, China |
| sorbitol | Sangon Biotech, China |
| uridine-5’-phosphate (UMP) | Sigma, China |
| superfidelity enzyme | Vazyme Biotech, China |
| FastDigest HindIII and XbaI | Thermo Fisher Scientific, USA |
| DNA purification kit | Ayxgen, USA |
| 2 × Basic Assembly Mix | TransGen Biotech, China |
| *Escherichia coli* DH5α | TransGen Biotech, China |
| *Agrobacterium* AGL1 | TransGen Biotech, China |
| TB Green® Premix EX Taq^TM^ | Vazyme Biotech, China |
| LSM880 laser scanning confocal microscope | Carl Zeiss, Germany |
| fluorescence microscope | Nikon, Japan |
| enhanced BCA protein assay kit | Beyotime, China |
| rapid PAGE gel preparation kit | Epizyme, China |
| anti-phosphorylated Pmk1 antibody | Cell Signaling Technology, USA |
| anti-non-phosphorylated Pmk1 antibody | Santa Cruz Biotechnology, USA |
| anti-phosphorylated Osm1 antibody | Cell Signaling Technology, USA |
| anti-GFP antibody | Huabio, China |
| anti-GAPDH antibody | Huabio, China |
| goat anti-rabbit/mouse IgG HRP | Huabio, China |
| enhanced chemiluminescence HRP kit | Fudebio Science, China |
| gel imaging analyzer | Bio-Rad, USA |
| Matchmaker gal4 two-hybrid system 3 | Clontech, USA |
| *E. coli* expression strain BL21 | TransGen Biotech, China |
| ultrasonic cell disruptor | Scientz, China |
| glutathione agarose beads | Invitrogen, USA |
| anti-GST antibody | Huabio, China |
| anti-Flag antibody | Huabio, China |

Figure S1


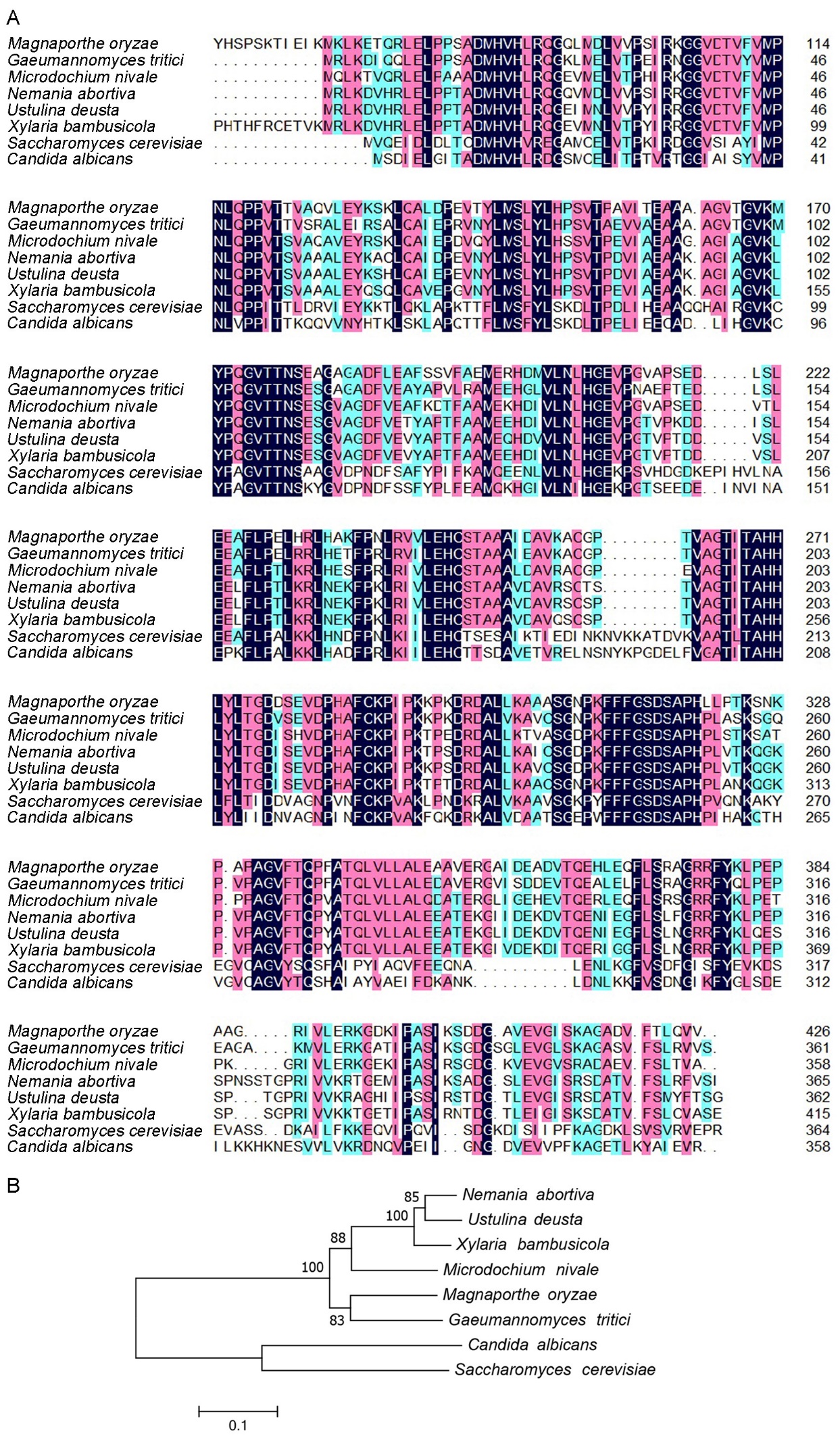


Figure S2


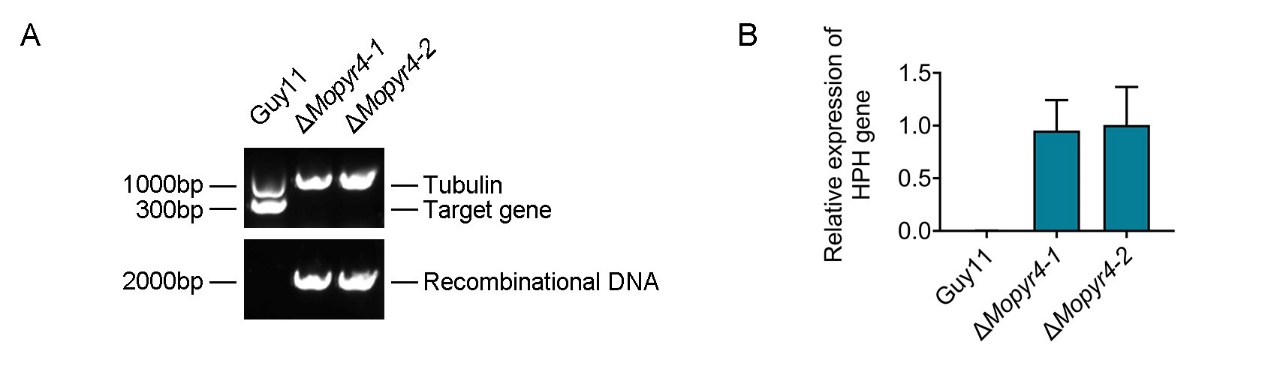


Figure S3


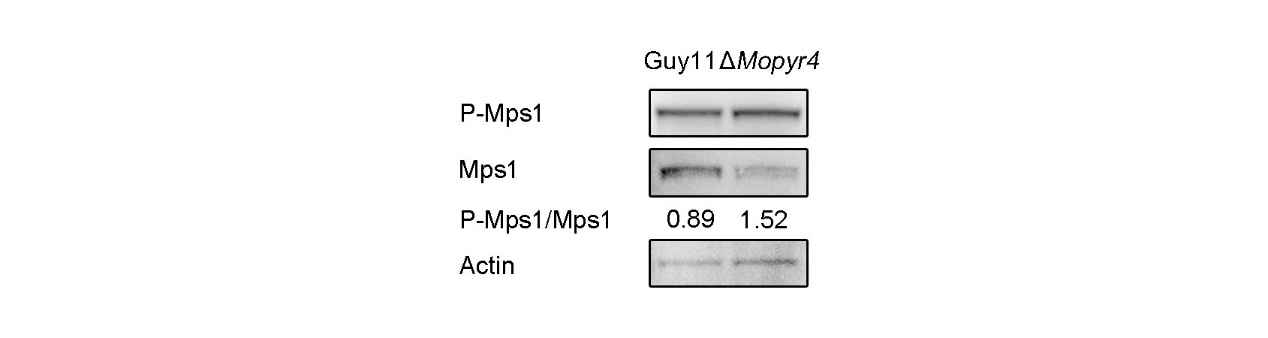

Supplement: Supplementary file 2 — Supplementary Material 2 [file 12964_2024_1741_MOESM2_ESM.docx]
